# Circulating SARS-CoV-2 spike N439K variants maintain fitness while evading antibody-mediated immunity

**DOI:** 10.1016/j.cell.2021.01.037

**Published:** 2021-03-04

**Authors:** Emma C. Thomson, Laura E. Rosen, James G. Shepherd, Roberto Spreafico, Ana da Silva Filipe, Jason A. Wojcechowskyj, Chris Davis, Luca Piccoli, David J. Pascall, Josh Dillen, Spyros Lytras, Nadine Czudnochowski, Rajiv Shah, Marcel Meury, Natasha Jesudason, Anna De Marco, Kathy Li, Jessica Bassi, Aine O’Toole, Dora Pinto, Rachel M. Colquhoun, Katja Culap, Ben Jackson, Fabrizia Zatta, Andrew Rambaut, Stefano Jaconi, Vattipally B. Sreenu, Jay Nix, Ivy Zhang, Ruth F. Jarrett, William G. Glass, Martina Beltramello, Kyriaki Nomikou, Matteo Pizzuto, Lily Tong, Elisabetta Cameroni, Tristan I. Croll, Natasha Johnson, Julia Di Iulio, Arthur Wickenhagen, Alessandro Ceschi, Aoife M. Harbison, Daniel Mair, Paolo Ferrari, Katherine Smollett, Federica Sallusto, Stephen Carmichael, Christian Garzoni, Jenna Nichols, Massimo Galli, Joseph Hughes, Agostino Riva, Antonia Ho, Marco Schiuma, Malcolm G. Semple, Peter J.M. Openshaw, Elisa Fadda, J. Kenneth Baillie, John D. Chodera, The ISARIC4C Investigators, the COVID-19 Genomics UK (COG-UK) Consortium, Suzannah J. Rihn, Samantha J. Lycett, Herbert W. Virgin, Amalio Telenti, Davide Corti, David L. Robertson, Gyorgy Snell

**Affiliations:** 1 MRC-University of Glasgow Centre for Virus Research, University of Glasgow, Glasgow G61 1QH, UK; 2 Department of Clinical Research, London School of Hygiene and Tropical Medicine, London WC1E 7HT, UK; 3 Vir Biotechnology, San Francisco, CA 94158, USA; 4 Humabs Biomed SA, a subsidiary of Vir Biotechnology, 6500 Bellinzona, Switzerland; 5 Institute of Biodiversity, Animal Health and Comparative Medicine, Boyd Orr Centre for Population and Ecosystem Health, University of Glasgow, Glasgow G61 1QH, UK; 6 Institute of Evolutionary Biology, University of Edinburgh, Edinburgh EH9 3FL, UK; 7 Molecular Biology Consortium, Advanced Light Source, Lawrence Berkeley National Laboratory, Berkeley, CA 94720, USA; 8 Computational and Systems Biology Program, Sloan Kettering Institute, Memorial Sloan Kettering Cancer Center, New York, NY 10065, USA; 9 Tri-Institutional PhD Program in Computational Biology and Medicine, Weill Cornell Graduate School of Medical Sciences, New York, NY 10065, USA; 10 Cambridge Institute for Medical Research, Department of Haematology, University of Cambridge, Cambridge CB2 0XY, UK; 11 Faculty of Biomedical Sciences, Università della Svizzera italiana, 6900 Lugano, Switzerland; 12 Division of Clinical Pharmacology and Toxicology, Institute of Pharmacological Sciences of Southern Switzerland, Ente Ospedaliero Cantonale, 6900 Lugano, Switzerland; 13 Department of Clinical Pharmacology and Toxicology, University Hospital Zurich, 8091 Zurich, Switzerland; 14 Department of Chemistry and Hamilton Institute, Maynooth University, Maynooth, Ireland; 15 Department of Nephrology, Ospedale Civico Lugano, Ente Ospedaliero Cantonale, 6900 Lugano, Switzerland; 16 Prince of Wales Hospital Clinical School, University of New South Wales, Sydney, NSW 2052, Australia; 17 Institute for Research in Biomedicine, Università della Svizzera italiana, 6500 Bellinzona, Switzerland; 18 ETH Institute of Microbiology, ETH Zurich, 8093 Zürich, Switzerland; 19 Clinic of Internal Medicine and Infectious Diseases, Clinica Luganese Moncucco, 6900 Lugano, Switzerland; 20 III Division of Infectious Diseases, ASST Fatebenefratelli Sacco, Luigi Sacco Hospital, 20157 Milan, Italy; 21 NIHR Health Protection Research Unit in Emerging and Zoonotic Infections, Institute of Infection, Veterinary and Ecological Sciences, Faculty of Health and Life Sciences, University of Liverpool, Liverpool L69 7BE, UK; 22 Respiratory Medicine, Alder Hey Children’s Hospital, Liverpool L12 2AP, UK; 23 National Heart and Lung Institute, Imperial College London, London SW3 6LY, UK; 24 The Roslin Institute, University of Edinburgh, Edinburgh EH25 9RG, UK; 25 Intensive Care Unit, Royal Infirmary Edinburgh, Edinburgh EH16 4SA, UK; 26 ISARIC4C Investigators; 27 https://www.cogconsortium.uk; 28 Washington University School of Medicine, Saint Louis, MO 63110, USA

**Keywords:** SARS-CoV-2, COVID-19, mutation, N439K, variant, Spike, receptor binding motif, monoclonal antibody escape, protein structure

## Abstract

SARS-CoV-2 can mutate and evade immunity, with consequences for efficacy of emerging vaccines and antibody therapeutics. Here, we demonstrate that the immunodominant SARS-CoV-2 spike (S) receptor binding motif (RBM) is a highly variable region of S and provide epidemiological, clinical, and molecular characterization of a prevalent, sentinel RBM mutation, N439K. We demonstrate N439K S protein has enhanced binding affinity to the hACE2 receptor, and N439K viruses have similar *in vitro* replication fitness and cause infections with similar clinical outcomes as compared to wild type. We show the N439K mutation confers resistance against several neutralizing monoclonal antibodies, including one authorized for emergency use by the US Food and Drug Administration (FDA), and reduces the activity of some polyclonal sera from persons recovered from infection. Immune evasion mutations that maintain virulence and fitness such as N439K can emerge within SARS-CoV-2 S, highlighting the need for ongoing molecular surveillance to guide development and usage of vaccines and therapeutics.

## Highlights

• The receptor-binding motif (RBM) is a highly variable region of SARS-CoV-2 spike
• RBM mutation N439K has emerged independently in multiple lineages
• N439K increases spike affinity for hACE2; viral fitness and disease are unchanged
• N439K confers resistance to several mAbs and escapes some polyclonal responses

## Graphical Abstract

**Figure:**
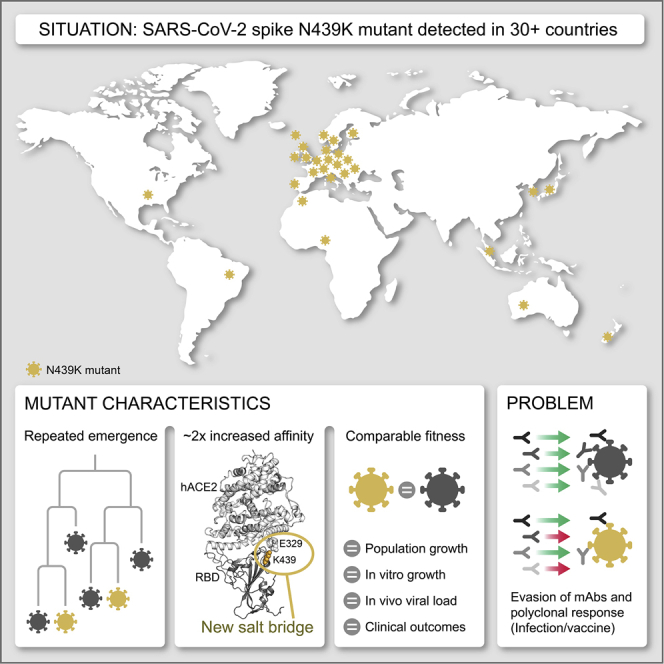

## Teaser

Epidemiological, clinical, molecular, and structural characterization of the N439K mutation in the SARS-CoV-2 spike receptor binding motif demonstrates that it results in similar viral fitness compared to wild-type while conferring resistance against some neutralizing monoclonal antibodies and reducing the activity of some polyclonal antibody responses.

## Introduction

SARS-CoV-2, the cause of COVID-19, emerged in late 2019 and expanded globally, resulting in over 82 million confirmed cases as of the end of 2020. Molecular epidemiology studies across the world have generated over 330,000 viral genomic sequences, shared with unprecedented speed via the GISAID Initiative (https://gisaid.org). These data are essential for monitoring virus transmission and spread (Meredith et al., 2020). Of special interest is the evolution of the SARS-CoV-2 surface protein, spike (S), which is responsible for viral entry via its interaction with the human angiotensin-converting enzyme 2 (hACE2) receptor on host cells. The S protein is the target of neutralizing antibodies generated by infection (Jiang et al., 2020) or vaccination (Folegatti et al., 2020; Jackson et al., 2020; Keech et al., 2020) as well as monoclonal antibody (mAb) drugs currently in clinical trials and/or approved for Emergency Use Authorization (EUA) by the US Food and Drug Administration (FDA) (Chen et al., 2021; Hansen et al., 2020; Jones et al., 2020; Pinto et al., 2020).

A SARS-CoV-2 S amino acid change, D614G, is now dominant in most places around the globe (Korber et al., 2020). Studies *in vitro* indicate that this mutation confers greater infectivity while molecular epidemiology correlates it with an increase in transmissibility with no evidence to date for increased virulence (Hou et al., 2020; Hu et al., 2020; Korber et al., 2020; Volz et al., 2021; Zhang et al., 2020). Amino acid 614 is located outside the receptor binding domain (RBD) of S, the domain targeted by 90% of neutralizing antibody activity in serum of SARS-CoV-2 survivors (Piccoli et al., 2020). Initial studies suggest that D614G viruses exhibit increased sensitivity to neutralizing antibodies, likely due to the effect of the mutation on the molecular dynamics of the S protein (Hou et al., 2020; Weissman et al., 2021; Yurkovetskiy et al., 2020). Therefore, this now dominant variant is unlikely to jeopardize natural or vaccine-derived antibody-mediated immunity generated in response to D614 S protein.

The low numbers of novel mutations reaching high frequency in sequenced SARS-CoV-2 genomes relates to the moderate intrinsic error rate of SARS-CoV-2 RNA replication (Li et al., 2020c; Robson et al., 2020). Nevertheless, the increasing number of infected individuals and the large reservoir of hosts susceptible to infection increase the likelihood that novel variants that impact vaccine and therapeutic development will emerge and spread by chance. Moreover, the full impact of immune selection, which can drive variant selection, has not yet influenced the pandemic, because herd immunity has not been attained. As population immunity increases and vaccines are deployed at scale, this will very likely change. The potential for circulating viral variants to derail promising vaccine or antibody-based prophylactics or treatments, even in the absence of selective pressure from the drug or vaccine, is demonstrated by the failure of a phase III clinical trial of a mAb targeting the respiratory syncytial virus (Simões et al., 2020) and the need for new influenza vaccines on a yearly basis. It is therefore critical to understand whether and how SARS-CoV-2 may evolve to evade antibody-dependent immunity.

Here, we examine the immunodominant SARS-CoV-2 receptor binding motif (RBM), the primary target of the neutralizing Ab response within the RBD (Piccoli et al., 2020), and find it to be a highly variable region of the S protein in circulating viruses. To understand the implications of this structural plasticity, which could allow the RBD to accommodate amino acids changes that could contribute to immune evasion, we defined the clinical and epidemiological impact, molecular features, and immune response to the RBM mutation N439K. This amino acid replacement has arisen independently multiple times, and in two cases formed lineages of more than 500 sequences. As of January 6, 2021, it was observed in 34 countries and was the second most commonly observed RBD mutation worldwide, and the sixth most common S mutation. We find that the N439K mutation results in enhanced RBD affinity for hACE2, it is associated with a similar clinical spectrum of disease and slightly higher viral loads *in vivo* compared to viruses with the wild-type (WT) N439 residue, and it results in immune escape from polyclonal sera from a proportion of recovered individuals and some neutralizing mAbs.

N439K provides a sentinel example of immune escape, indicating that RBM variants must be evaluated when considering vaccines and the therapeutic or prophylactic use of mAbs. Long-term control of the pandemic with vaccines will require systematic monitoring of immune escape variants and may require new vaccine preparations that address the variants circulating globally.

## Results

### The RBM is a variable region of the SARS-CoV-2 S protein

Competing pressures influence the evolution of the S RBM. First, the RBM mediates viral entry (Shang et al., 2020; Walls et al., 2020; Wrapp et al., 2020b) and therefore must maintain sufficient affinity to engage the entry receptor hACE2. Second, it is a major target of neutralizing antibodies (Piccoli et al., 2020; Robbiani et al., 2020; Rogers et al., 2020; Wec et al., 2020) and so would be a primary location for the emergence of immune escape mutations. We set out to understand these competing pressures by evaluating the landscape of RBM sequence divergence observed in circulating SARS-CoV-2 variants and in other viruses of the *Sarbecovirus* lineage.

**Figure 1:**
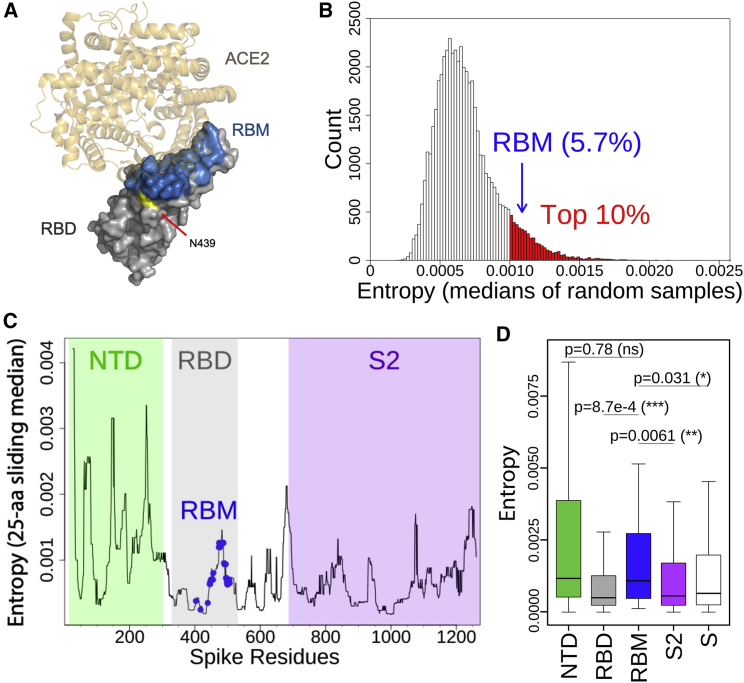
The RBM exhibits significant natural diversity in circulating SARS-CoV-2 viruses SARS-CoV-2 variants (retrieved from CoV-GLUE) are based on 209,239 high-quality sequences downloaded from GISAID on November 30, 2020. (A) Structure of the SARS-CoV-2 RBD-hACE2 complex (PDB: 6M0J) highlighting the RBM (blue) and residue N439 (yellow). (B) Thirty-four residues (the size of the RBM) were randomly sampled without replacement 50,000 times from the mature S protein (excluding the RBM). Median entropies were computed for each draw. The resulting 50,000 median entropies were used to build the entropy distribution of residues other than the RBM. The top 10% medians are highlighted in red. The median entropy of RBM residues was compared with the non-RBM entropy distribution to determine the variability of the RBM relative to non-RBM residues. To allow for a fair comparison, sampling was performed without enforcing residue contiguity, as the RBM is not contiguous in sequence space. Therefore, in any given sample, residues are unlikely to share any functional relationship. (C) Per-residue entropies of the mature S protein were smoothed by plotting medians of a 25-aa center-aligned sliding window. Smoothing allows visualizing local peaks of variability. The RBM residues and the NTD, RBD, and S2 domains are highlighted. Due to the non-contiguous nature of the RBM in sequence space, the sliding window median at RBM residues is diluted by neighboring non-RBM residues. (D) Boxplot of per-residue entropies in four S domains (or full mature S protein). The lower and upper hinges correspond to the first and third quartiles. The lower/upper whiskers extend from the hinge to the smallest/largest value no further than 1.5 times the inter-quartile range. Outliers beyond the end of the whiskers are not plotted but are retained for statistical testing. Pairwise comparisons by Mann-Whitney U tests. p value thresholds are 0.05 (^∗^), 0.01 (^∗∗^) and 0.001 (^∗∗∗^); ns, not significant. See also Figures S1 and S2.

**Figure S1:**
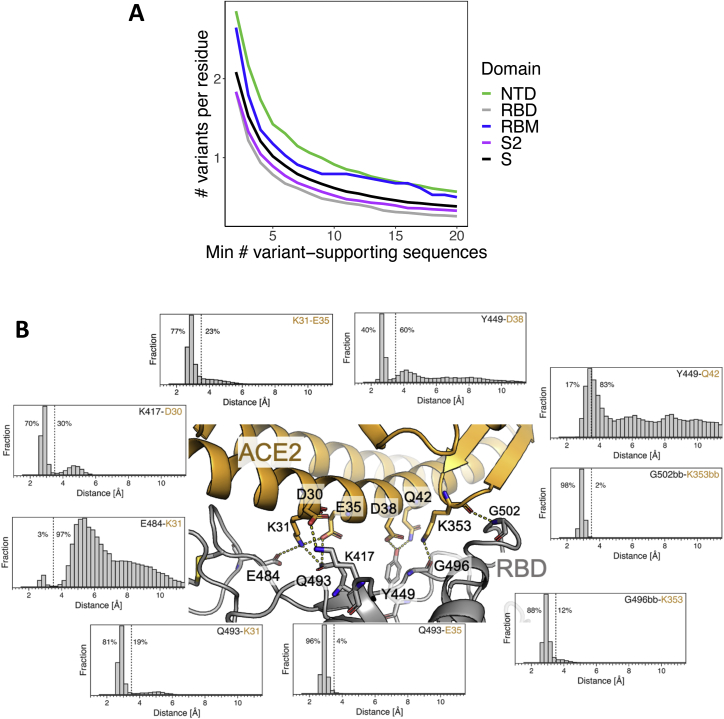
High RBM variability in deposited SARS-CoV-2 sequences is consistent with a dynamic RBD:hACE2 binding interface, related to Figures 1 and 2 (A) Number of observed variants in four S domains (or full mature S protein) normalized by the total number of residues in each domain, where the number of observed isolates required to call a variant is varied along the x axis. (B) Distributions of distances observed for RBD (gray):hACE2 (gold) residue pairs: K417-D30, E484-K31, Q493-K31, Q493-E35, G496bb-K353, G502bb-K353bb, Y449-Q42, Y449-D38, K31-E35 (bb = backbone interaction). RBD:ACE2 residue pairs were chosen based on RBM residues with high binding energies as determined by the binding energy % column (green) in Figure 2. Distances were computed every 2.5 ns from 118.7 μs of molecular dynamics simulation data. Dashed lines indicate a distance of 3.5 Å and the percentage of distances below and above 3.5 Å are annotated to the left and right of the lines, respectively.

**Figure S2:**
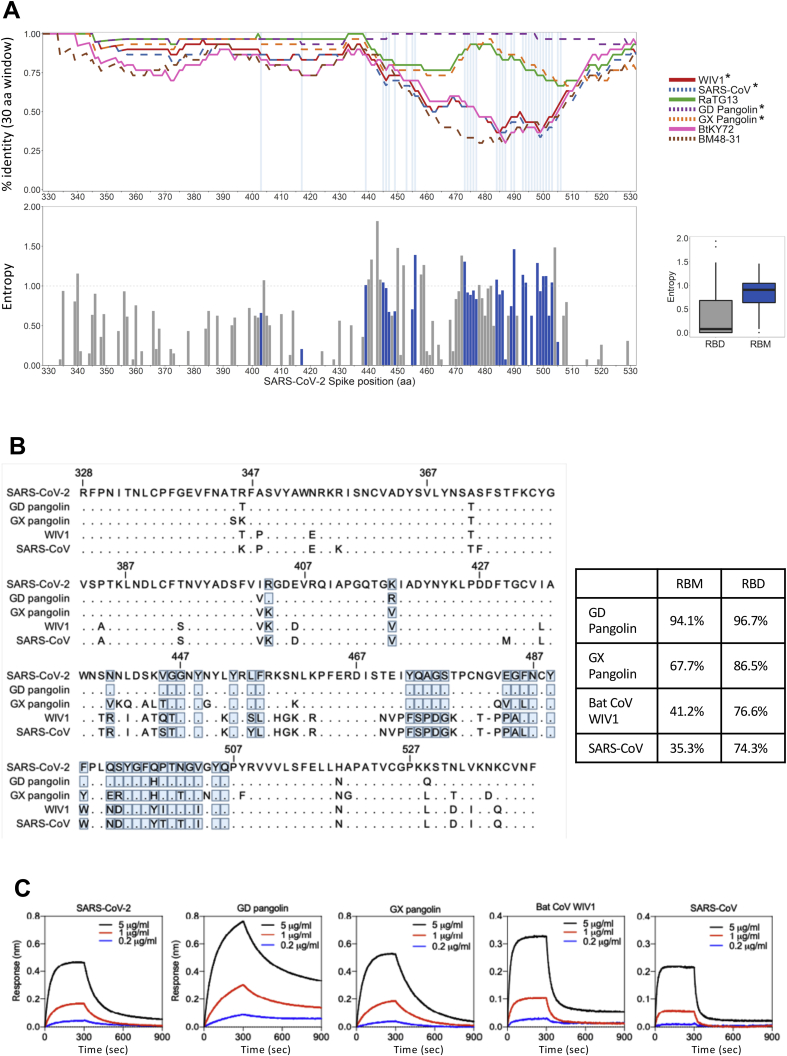
RBDs from bat and pangolin *Sarbecovirus* isolates bind to hACE2 despite RBM divergence, related to Figures 1 and 2 (A) Top – Percent identity to SARS-CoV-2 using a sliding window size of 30 amino acids for seven related *Sarbecoviruses* (see figure key, ^∗^: viruses which bind to hACE2) across the RBD region of the Spike protein. Bottom – Site-specific entropy plot across the RBD protein alignment of SARS-CoV-2 and 68 related viruses (Table S1). Sites constituting the RBM are annotated in blue; the x axis refers to absolute positions in the SARS-CoV-2 Spike protein sequence. Right – boxplot of site-specific entropy values for the RBM sites (blue) and the full RBD (gray). (B) Sequence alignment (left) and identity for RBM and RBD (right) to SARS-CoV-2 of the RBD sequences showing binding to hACE2. RBM residues indicated by blue boxes. (C) Binding of hACE2 to human, pangolin, and bat *Sarbecovirus* RBDs by BLI. Bat CoV RaTG13, Bat CoVs ZC45, BtKY72 and BGR2008 have also been tested and did not bind hACE2.

We used re-refined published X-ray structures of SARS-CoV and SARS-CoV-2 RBD:hACE2 complexes (Lan et al., 2020; Li et al., 2005) to define the RBM residues using a 6 Å distance cutoff (Figures 1A–1D and S1A, 2). We evaluated SARS-CoV-2 genomic sequences deposited in GISAID as of November 30th, 2020 and observed a high number of variants occurring in the RBM. To understand how the variability of the RBM compares to the variability of the entire RBD and the whole S protein, we evaluated well-defined S protein domains: within S1, the N-terminal domain (NTD) and the RBD (further split into RBM and non-RBM), and the S2 domain. Analysis of entropy, which estimates sequence variability at a given position in a protein alignment, identified the RBM as a highly variable region of the RBD and of the entire S protein (Figures 1B–1D), with a median entropy within the top 10% of equivalently sized sets of randomly sampled residues (Figure 1B). This result is confirmed by an analysis of sequence variability that is not weighted by total counts of each variant, thereby capturing the diversity of circulating variants with mitigated bias toward oversampled variants (Figure S1A).

**Figure 2:**
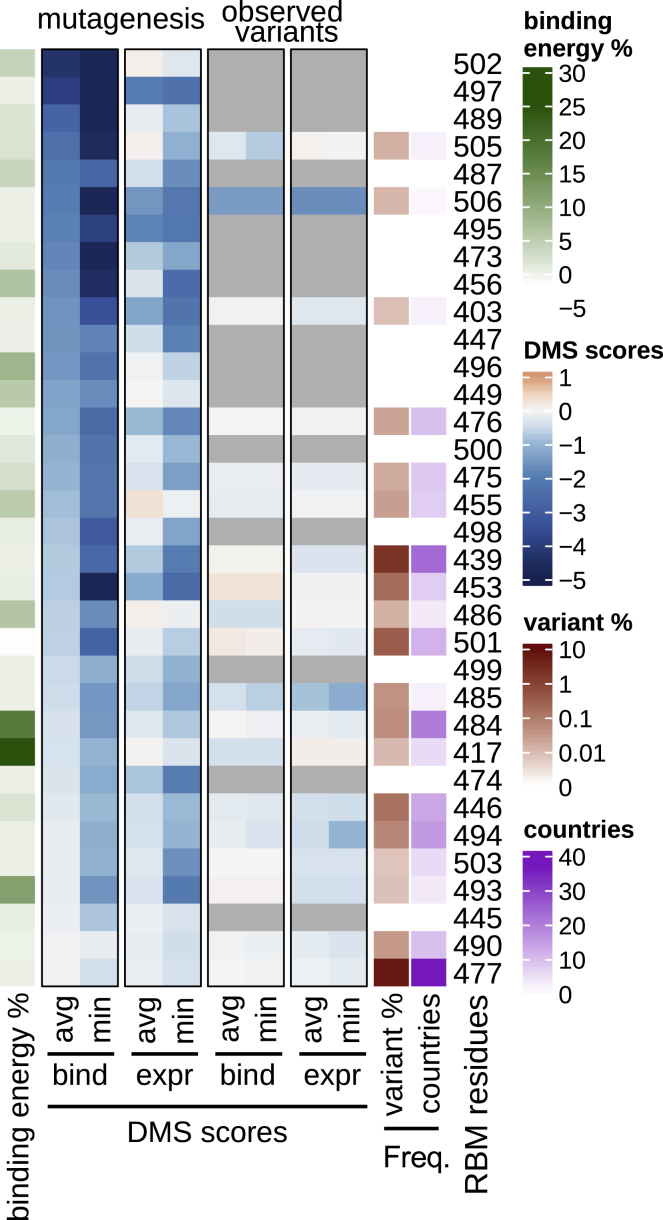
RBM functional constraints compared to RBM natural diversity Each residue in the RBM is annotated by several metrics, depicted as a heatmap. DMS scores: outlined in black boxes (center) are summaries of hACE2 binding and RBD expression deep mutational scanning (DMS) experimental results (Starr et al., 2020b). DMS score is the binding or expression fold change of a variant over WT on a log10 scale (red indicating improvement and blue indicating loss as compared to WT). In the “mutagenesis” columns, DMS results are given for each residue as either the minimum (most disruptive variant) or the average score across all possible variants of a residue, except for the reference residue and the stop codon. In the “observed variants” columns, minimum and average scores are computed only across variants that have been observed in GISAID (same set of sequences as used for Figure 1). When no natural variants have been observed, cells are gray. Data were sorted on the leftmost DMS column. Frequency: each RBM position is annotated with the frequency of non-reference amino acids in deposited sequences (darker red indicating higher frequency; at least 1 supporting sequence per 25,000 deposited sequences is required to call a variant). The number of countries in which variants have been observed is also annotated (darker purple indicating more countries). Binding energy: a re-refined SARS-CoV-2 RBD:hACE2 complex X-ray structure (PDB: 6M0J) was used to determine the approximate, decomposed binding free energy associated with each RBM residue. Results for each RBM residue are expressed as a percentage of the total binding interface interaction energy (darker green indicating stronger contribution to the binding energy). See also Figures S1 and S2.

To understand constraints on RBM variability, we evaluated the published deep mutational scanning (DMS) dataset of the RBD (Starr et al., 2020b) and compared it to sequences of circulating viruses. The DMS data define the effect of each possible single amino acid change on both expression of the RBD and its capacity to bind hACE2. For each position in the RBM, we compared the DMS results for all amino acid replacements at that position versus only changes that have been observed in circulating SARS-CoV-2 variants (Figure 2). A subset of residues shows the largest loss of hACE2 binding on mutation (top ∼1/3 of RBM residues in Figure 2) and, as would be expected, few natural occurrences of mutations at these residues have been observed to be circulating. However, for the majority of the RBM (bottom ∼2/3 of RBM residues in Figure 2), variation in circulating virus sequences confirms the tolerance to mutation predicted by the DMS data.

To further assess the ability of the RBM to accommodate mutations without disrupting hACE2 binding, we examined the structural dynamics and energetics of the RBM:hACE2 binding interface. We performed an approximate, residue-level decomposition of binding free energy based on the RBD:hACE2 complex X-ray structure (green in Figure 2) as well as molecular dynamics simulations of the complex, resulting in ∼118 μs of aggregate simulation data (Figure S1B). Consistent with expectation, the two residues with the highest variant frequency (S477 and N439) contribute weakly to the binding energy (Figure 2). Surprisingly, the two RBM residues with the strongest interactions with hACE2 based on the X-ray structure (K417 and E484, dark green in Figure 2) were not highly conserved (variant % in red, Figure 2), with ∼10-fold more variants for E484. This could be explained by results from the molecular dynamics simulation: K417 formed close interactions with hACE2 70% of the simulation time, while E484 only 3% of the time (Figure S1B). The low percent for E484 is also consistent with the non-conservative amino acid replacements observed for circulating variants (e.g., the most common E484 substitution is currently E484K), with a positively charged lysine substituting for the negatively charged glutamate. Overall, these results demonstrate that the RBM has a high degree of structural plasticity whereby it is able to accommodate amino acid changes without disrupting hACE2 binding.

Evolutionary analysis of the *Sarbecovirus* subgenus provides further support for RBM plasticity (Boni et al., 2020; Li et al., 2020b; Rambaut et al., 2020). The SARS-CoV RBM is highly divergent from the SARS-CoV-2 RBM (Figures S2A and S2B) while maintaining hACE2 binding affinity. Additionally, there are many sequence changes in the RBM across a panel of related coronaviruses from animal isolates (Figures S2A and S2B; Table S1). To determine the ability of members of the *Sarbecovirus* lineage to bind hACE2, we produced nine recombinant RBD proteins corresponding to seven animal isolates, SARS-CoV-2, and SARS-CoV, and evaluated their binding to recombinant hACE2 (Figure S2C). We found that three of the RBDs from animal isolates showed strong affinity for hACE2: GD Pangolin, which has a highly similar RBM to SARS-CoV-2, GX Pangolin, which has a more divergent RBM, and Bat CoV WIV1 which is highly divergent (Figures S2A and S2B). This further indicates that the RBM is structurally plastic, retaining binding with hACE2 as a receptor despite changes to sequence. Given this plasticity, we next considered whether an RBM variant can lead to immune evasion while retaining virulence.

### Phylogenetic analysis of the prevalent SARS-CoV-2 RBM mutation N439K

**Figure 3:**
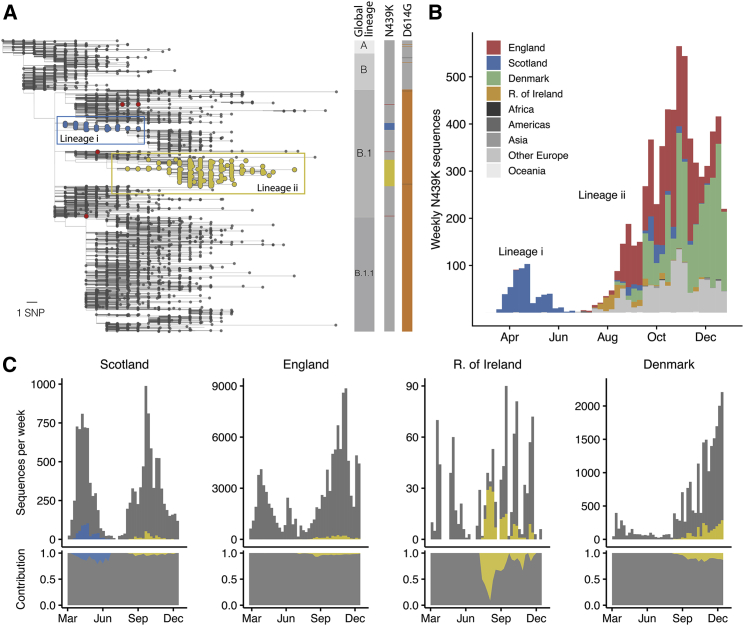
The N439K RBM mutation has arisen independently multiple times, twice forming significant lineages (A) Phylogenetic tree (de-duplicated and down-sampled) showing the relationship among representative global SARS-CoV-2 variants, with N439K variants highlighted in color. Two significant N439K lineages, one in Scotland (>500 sequences, blue circles) and one in 32 countries (>6,000 sequences, yellow circles) were detected as of January 6, 2021. The N439K mutation has also emerged independently on at least seven occasions (red circles show four of these) bringing the total country count to 34. Vertical bars indicate global lineage, the presence of N439K (same colors as tree), D614G (orange) or D614N (dark gray). The scale bar corresponds to a single nucleotide polymorphism (SNP). (B) Frequency of N439K variants relative to sampling time and their geographical area of occurrence (see key): Africa (Morocco, Nigeria), Americas (Brazil, USA), Asia (Japan, Singapore, South Korea), the European countries Denmark, England, Republic of Ireland and Scotland and other European countries (Belgium, Bosnia-Herzegovina, Croatia, Czech Republic, Faroe Islands, Finland, France, Germany, Hungary, Italy, Luxembourg, Netherlands, Northern Ireland, Norway, Poland, Romania, Slovakia, Sweden, Switzerland, Wales), and Oceania (Australia, New Zealand). The prominent light gray bars correspond to other European countries. See Table S2 for total numbers for each country. (C) Frequency of the two N439K lineages (same colors as A) over time relative to all sequences for that country (gray) and their normalized contributions (lower panels) in Scotland, England, Republic of Ireland, and Denmark. See also Figure S3.

N439K is a prevalent RBM mutation (the second most common mutation in the RBD through the end of 2020) which was first sampled in March 2020 in Scotland from lineage B.1 (Rambaut et al., 2020) on the background of D614G. Using phylogenetic analysis, we determined that the earliest reported N439K sequences represented a single SARS-CoV-2 lineage (Figure 3A) that increased in frequency to 542 sequences in Scotland by June 20, 2020 (∼10% of the available Scottish viral genome sequences for this time period). Subsequently, numbers of N439K and all other variants decreased in Scotland concurrent with control of the pandemic after initiation of stringent public health measures, with this specific N439K lineage (designated here as lineage i) not being detected since June 2020 (Figures 3B and 3C). However, the N439K mutation appears in >6,000 additional sequences in the GISAID database as of January 6, 2021. Our analysis demonstrates that the majority of these sequences represent a second, independent lineage (designated lineage ii) which was first sampled in Romania on May 13, 2020, then Norway on June 23, 2020, and is now detected to be circulating in 32 countries (Figures 3A–3C). N439K lineages i and ii have recently received the lineage designations B.1.141 and B.1.258, respectively (Rambaut et al., 2020). We also observe at least seven instances of the N439K mutation that have arisen independently of these two large lineages, including again in the United States in at least four linked infections, and in Brazil and Nigeria where no lineage ii/B.1.258 has been observed, resulting in a total of 34 countries where N439K has been detected to date (Figures 3A and 3B).

Sequence counts are heavily influenced by sampling frequency, which varies widely between countries, and N439K as a percentage of total sequences appears low: as of January 6, 2021, there have been 6,868 N439K observations in GISAID, 2% out of ∼290,000 SARS-CoV-2 genome sequences for the 34 countries where this mutation has been detected (Table S2). Nevertheless, when comparing the percentage of N439K sequences over time in countries with sufficient data, the proportion can be significant: ∼10% in Scotland from March to June 2020 and ∼10% in Denmark from August to December 2020, both countries with high sequencing rates, and ∼13% in Ireland from July to December 2020, where regional coverage is reasonable, but the sequencing rate is lower (Figure 3C). Importantly, on the scale of a pandemic, small proportions correspond to large numbers of infections. If the proportion of N439K sequences in each country predicts what proportion of its confirmed infections are associated with N439K variants, then N439K variants correspond to ∼764,000 of the confirmed SARS-CoV-2 infections as of January 6, 2021 (Table S2). If detected cases represent 5%–33% of true infections, as has been estimated for the United States (Wu et al., 2020b), then a very rough approximation of the actual cumulative number of N439K-associated infections would be in the range of 2–15 million.

Overall, the spread of N439K to at least 34 countries is concerning, as is its repeated independent emergence. At the nucleotide level, all N439K variants to date have arisen from the same mutation: a C-to-A transversion in the third codon position. Interestingly, 4,209 of sequences in lineage ii/B.1.258 also carry the S 69-70 deletion that has occurred independently multiple times in the pandemic and most notably with the Y453F amino acid replacement associated with mink infections (Oude Munnink et al., 2021). In both cases the 69-70 deletion mutation has arisen subsequent to the RBM mutation and then been retained in all subsequent variants. This deletion has also been recently reported to provide an escape for NTD-specific neutralizing antibodies (McCarthy et al., 2021). Very recently, this deletion has also been observed to co-occur with another RBM mutation, N501Y (Volz et al., 2021).

**Figure S3:**
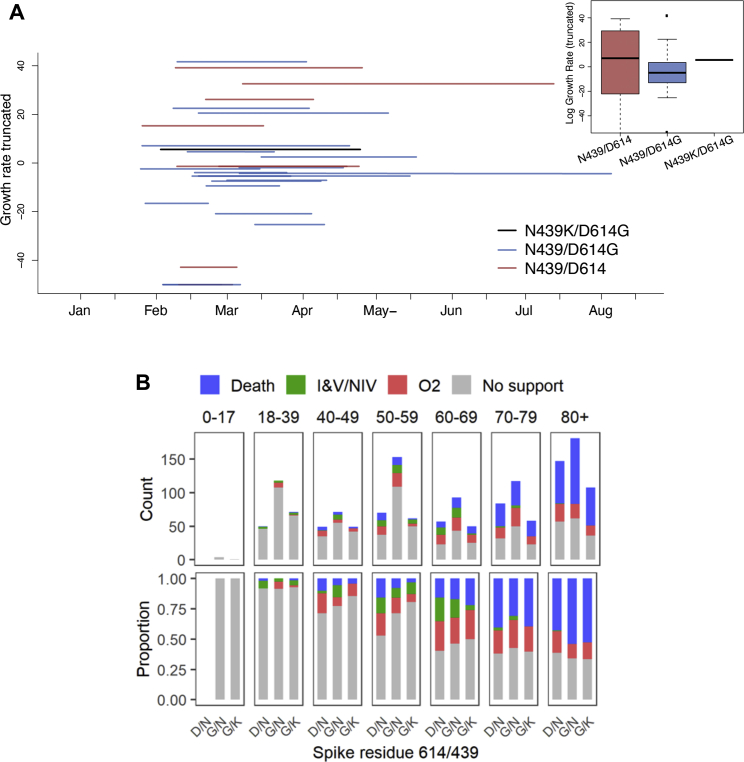
Virological and clinical results stratified by positions 439 and 614, related to Figures 3 and 5 (A) Phylodynamic analysis showing lineage growth rates relative to sampling times for UK lineages in Scotland. Data used for analysis were sampled between Feb 28, 2020 and Aug 18, 2020 (see STAR methods and http://sars2.cvr.gla.ac.uk/RiseFallScotCOVID/). The Scottish N439K lineage i (which co-occurs with D614G) is indicated in black along with whether wild-type N439 lineages are D614 (red) or D614G (blue). The inset shows a boxplot for the distributions of these genotypes. Note, only the growth rates between −50 and 50 are plotted. (B) Comparison of clinical severity between D614/N439, D614G/N439 and D614G/N439K genotypes by patient age group for 1591 patients whose diagnostic samples were sequenced. Ordinal scale scored by oxygen requirement: 1. No respiratory support, 2: Supplemental oxygen, 3: Invasive or non-invasive ventilation or oxygen delivery by high flow nasal cannulae, 4: Death.

Because there is concern that mutations with high prevalence may have increased virus transmissibility, we next evaluated whether any difference could be detected in the rate of spread of the N439K lineages as compared to other lineages. Because Scotland has a high sampling frequency for its population size (Table S2), it is possible to calculate a growth rate for N439K lineage i based on a comparison with other Scottish lineages (see STAR methods and http://sars2.cvr.gla.ac.uk/RiseFallScotCOVID/). We find that while the N439K/D614G lineage is one of the largest to emerge in Scotland, its growth rate is similar to the median N439/D614 or N439/D614G WT growth rates, with no evidence for a faster growth conferred by the N439K mutation (Figure S3A).

### N439K RBD forms a new interaction with hACE2 and has enhanced hACE2 affinity

**Figure 4:**
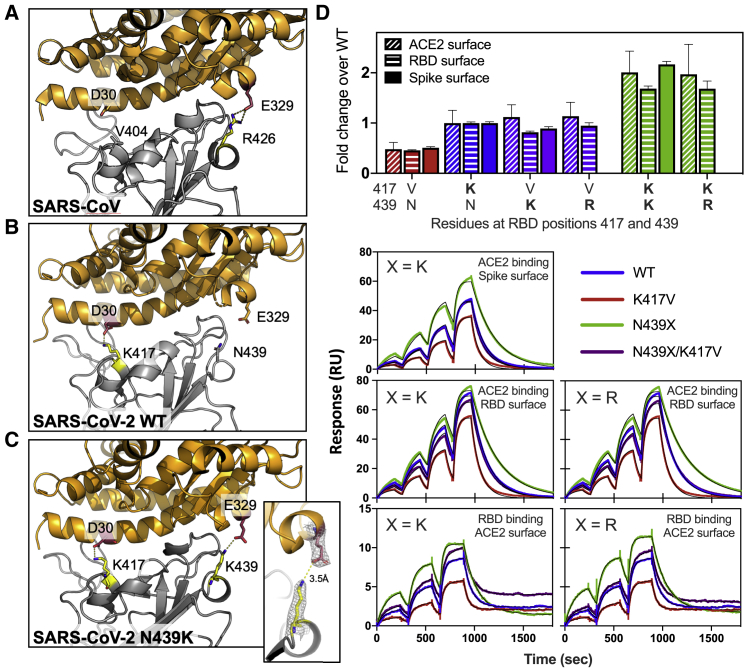
N439K creates a new RBD:hACE2 salt bridge and enhances RBD:hACE2 affinity (A–C) X-ray structures of the SARS-CoV (A), SARS-CoV-2 WT (B), and SARS-CoV-2 N439K (C) RBD in complex with hACE2 (based on 2AJF, 6M0J, and current work, respectively). Select interface residues are shown as sticks. hACE2 is shown in orange and RBD in gray. The inset in (C) shows the 2Fo-Fc electron density contoured at 1σ for the K439-E329 salt bridge. (D) Binding affinity of RBD and Spike variants for hACE2 measured by surface plasmon resonance. Monomeric hACE2 is injected successively at 11, 33, 100, and 300 nM onto surface-captured spike extracellular domain (ECD) or RBD; alternately, RBD is injected successively at 3.1, 12.5, and 50 nM onto surface-captured hACE2. All spike ECD contain the D614G mutation. Bar graph: affinity measurements (averages of 3–4 replicates) expressed as a fold change relative to WT binding within each experiment format, where >1 indicates improved binding (smaller K_D_) relative to WT. WT K_D_ values measured as: 95 ± 1.6 nM (Spike surface), 63 ± 1.0 nM (RBD surface), 19 ± 3.3 nM (hACE2 surface); errors are SEM. See also Table S3.

In addition to its frequency and repeated emergence, the N439K mutation stood out from other circulating RBM mutations as having a plausible mechanism for maintenance of viral fitness. The equivalent position to N439K in the SARS-CoV RBM is also a positively charged amino acid (R426), which forms a salt bridge with hACE2 (Li et al., 2005) (Figure 4A). We therefore hypothesized that the N439K SARS-CoV-2 variant may form a similar salt bridge at the RBD-hACE2 interface (RBD N439K:hACE2 E329) (Figure 4B). We determined the X-ray structure of the N439K RBD in complex with hACE2 at 2.8 Å resolution and observed that this new interaction does indeed form (Figure 4C; Table S3). Because salt bridges can be strong non-covalent bonds, and therefore the N439K mutation plausibly adds a strong interaction at the binding interface, we hypothesized that the N439K variant has enhanced binding for hACE2.

To test this hypothesis, we used surface plasmon resonance (SPR) to evaluate binding of recombinant N439K S or RBD protein to recombinant hACE2. We also evaluated the N439R and K417V variants, each of which are found in SARS-CoV at these positions, and the latter of which would remove a salt bridge at the RBD:hACE2 interface. Across multiple assay formats, we found that the N439K and N439R variants exhibited an ∼2-fold enhanced binding affinity for hACE2 as compared to the original N439 variant (termed herein WT) (Figure 4D). The magnitude of this enhancement was paralleled by an ∼2-fold loss of binding affinity for the K417V variant relative to WT. Our data are in line with the DMS results (Starr et al., 2020b), which show a 2-fold loss of binding for K417V and no change for N439K/R, as the two assays are inherently different and the DMS data are much higher-throughput but lower sensitivity. We also tested the effect of the N439K/R and K417V mutations in combination. These double mutants swap one salt bridge at the hACE2 binding interface at RBD position 417 for one at position 439; we found they had an hACE2 affinity similar to the WT (Figure 4D).

Overall, these data indicate that acquisition of the N439K mutation enhances hACE2 binding, which could have implications *in vivo* in the context of infection and transmission. At a minimum, we found no evidence for any decreased success of N439K lineage i relative to other lineages present in Scotland at the same time (Figure S3A). The enhanced affinity could compensate for other mutations that would otherwise decrease binding (e.g., K417V), further highlighting the plasticity of the RBM and the need for surveillance.

### N439K SARS-CoV-2 maintains fitness and virulence

The enhanced hACE2 affinity conferred by the N439K mutation, its geographical emergence as independent lineages, as well as its prevalence among circulating viral isolates is consistent with no effect on viral fitness. We set out to directly examine N439K impact on viral fitness by evaluating clinical data and outcomes associated with virus carrying the N439K mutation versus WT N439, as well as by direct *in vitro* viral growth and competition. Clinical data including age, gender, date of diagnosis, hospitalization status, and mortality were collected prospectively, and sequencing was carried out in real time, as part of the Scottish strategy for COVID-19 surveillance.

**Figure 5:**
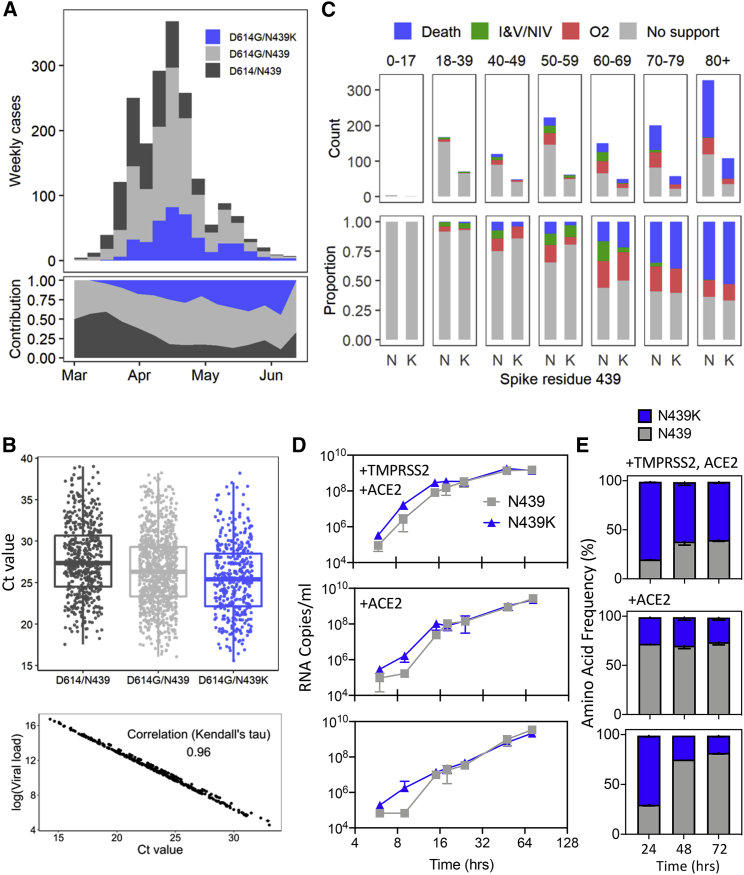
Clinical outcomes and virological evaluation of N439K lineage i indicate maintenance of fitness relative to WT virus (A) Epidemiological growth of the N439/D614, N439/D614G, or N439K/D614G virus in the National Health Service (NHS) Greater Glasgow and Clyde (GGC) Health Board area relative to sampling time in epidemiological (epi) weeks (top) and their relative contributions (bottom) for 1,918 patients whose diagnostic samples were sequenced. (B) Top: real-time PCR data for N439/D614, N439/D614G, and N439K/D614G groups, same patient population as in (A). The N439K genotype was associated with marginally lower Ct values than the N439 genotype (posterior mean Ct value difference between N439K/D614G and N439/D614G: −0.65, 95% CI: −1.22, −0.07). Bottom: correlation between Ct and quantitative viral load. (C) Severity of disease within NHS GGC for a subset of 1,591 patients. Ordinal scale scored by requirement for supplementary oxygen: (1) no respiratory support, (2) supplemental oxygen, (3) invasive or non-invasive ventilation or oxygen delivered by high-flow nasal cannula, and (4) death. Ordinal regression analysis indicated that the N439K viral genotype was associated with similar clinical outcomes compared to the N439 genotype (posterior mean of N439K/D614G genotype effect: 0.06, 95% CI: −1.21, 1.33). (D) Growth curves for GLA1 (N439/D614G) or GLA2 (N439K/D614G) virus isolates in Vero E6 cells with ACE2 and TMPRSS2 overexpression (+TMPRSS2 +ACE2), ACE2 overexpression (+ACE2), or no overexpression. Error bars are SD from three replicates. (E) Competition of GLA1 and GLA2 virus isolates for growth in Vero E6 cells with ACE2 and TMPRSS2 overexpression (+TMPRSS2 +ACE2), ACE2 overexpression (+ACE2), or no overexpression, after inoculation at a matched MOI. Quantification of each virus was performed by tracking the frequency of N439K within the spike gene using metagenomic NGS. Error bars are SD from three replicates. See also Figure S3 and Tables S4–S6.

We used qPCR to evaluate viral load (as measured by cycle threshold [Ct]) in 1,918 Scottish patients whose positive samples had been sequenced (Figures 5A and 5B). Variants were either N439K/D614G (n = 406), N439/D614G (n = 978), or ancestral (N439/D614) (n = 534). Our analysis found strong evidence that the N439K/D614G genotype was associated with marginally lower Ct than the N439/D614G genotype, even after controlling for confounders: age, sex, viral co-ancestry, and epidemic stage (mean Ct value difference between N439K/D614G and N439/D614G: −0.65, 95% confidence interval [CI]: −1.22, −0.07) (Figure 5B; Table S4). Assuming the PCR was 95% efficient, then a mean Ct difference of 0.65 would represent an RNA copy number increase of 1.54-fold in N439K/D614G relative to N439/D614G. Because Ct measurements were from multiple locations in Scotland, a sub-analysis of viral load using RNA standards was carried out with available samples. This analysis showed a near-complete correlation with Ct values (Figure 5B). D614G has previously been associated with higher viral loads/lower Ct values (Korber et al., 2020; Lorenzo-Redondo et al., 2020; Mueller et al., 2020; Volz et al., 2021); although our data suggest a similar trend in a naive analysis, when controlling for confounders (given above), we could not detect this effect (Table S4).

Clinical outcomes were also obtained for a subset of these patients (n = 1,591), who were scored for severity of disease based on oxygen requirement: (1) no respiratory support, (2) supplemental oxygen, (3) invasive or non-invasive ventilation or high flow nasal cannula, or (4) death (Figures 5C and S3B). The requirement for oxygen therapy or ventilation was collected retrospectively. Variant counts for the clinical outcome analysis were double mutant (N439K/D614G, n = 399), D614G mutants (with N439 WT, n = 735), or ancestral genotype (N439/D614, n = 457). Our ordinal regression indicated that the N439K/D614G viral genotype was associated with similar clinical outcomes compared to D614G or ancestral genotypes (posterior mean of N439K/D614G genotype effect: 0.06, 95% CI: −1.21, 1.33) (Table S5). All other results from the severity analysis were qualitatively similar to a previous analysis of the D614G mutation (Volz et al., 2021). These clinical outcome data indicate that the N439K virus is neither linked to an attenuated phenotype nor linked to increased severity.

We next experimentally tested growth of two representative SARS-CoV-2 isolates, GLA1 (N439) and GLA2 (N439K), both with the D614G background (Table S6). Culture was carried out for 72 h in Vero E6 cells with either hACE2 and TMPRSS2 overexpression, hACE2 overexpression, or no overexpression. There was no significant difference between the growth of these isolates after inoculation at multiplicities of infection (MOIs) of 0.005 and 0.01. The N439K variant replicated slightly faster initially after inoculation (Figure 5D). These experimental data indicate that the N439K mutation does not exhibit positive or negative effects on viral growth. To further assess fitness for replication in cultured cells, we carried out a cross-competition assay using inoculation of cells at a matched MOI followed by quantitation of N439 and N439K by metagenomic sequencing over time (Figure 5E). N439K demonstrated similar fitness as the WT N439 variant, with a slight fitness advantage for N439K in cells expressing TMPRSS2. Collectively, these results indicate that the N439K mutation results in viral fitness that is similar or possibly slightly improved relative to the WT N439 virus. These results may relate to the improved hACE2 affinity measured for the N439K RBD in the SPR binding assays, or could relate to additional mechanisms, such as changes to S density on the viral particle surface or changes to the conformational dynamics of the S protein.

### The N439K mutation promotes evasion of antibody-mediated immunity

**Figure 6:**
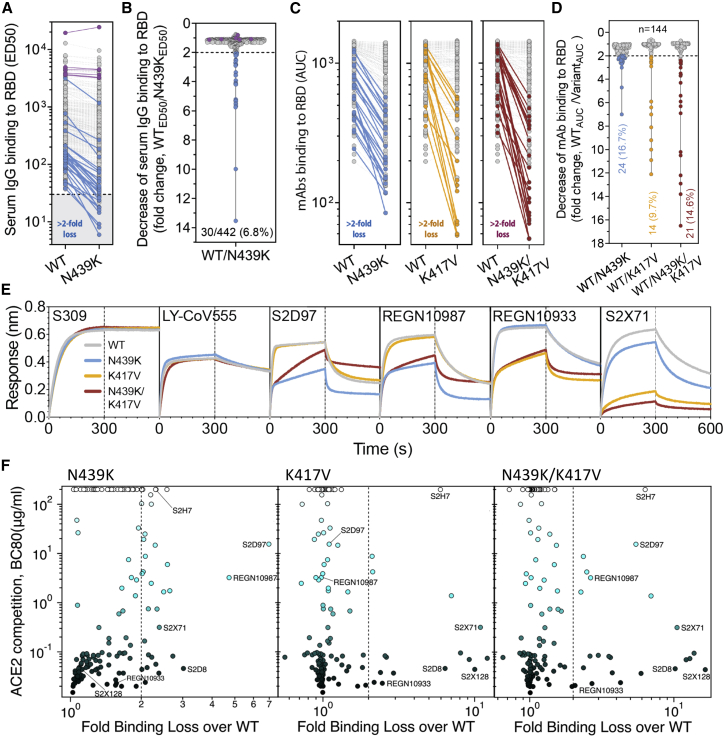
RBM variants exhibit escape from monoclonal antibodies and sera binding (A and B) Binding of serum and plasma samples from 442 SARS-CoV-2 infected individuals against WT and N439K RBD plotted as (A) ELISA ED_50_ for each RBD (cut-off for positive binding to WT set at 30) and (B) fold change relative to WT. Data shown are the average of two independent replicates (source data given in Data S1). Blue dots indicate sera with at least 2-fold loss of binding to the N439K RBD variant as compared to WT in both replicates. Purple dots indicate sera from individuals infected with SARS-CoV-2 N439K variant. (C and D) Binding of 140 mAbs from SARS-CoV-2 infected individuals and four clinical-stage or EUA-approved mAbs against WT, N439K, K417V, and N439K/K417V RBD, plotted as (C) ELISA AUC for each RBD and (D) fold change relative to WT. Data shown are the average of two independent replicates (source data given in Data S1). For all, the colored dots indicate mAbs demonstrating at least 2-fold loss of binding to the variant RBD as compared to WT (counted if the average of both replicates is at least 2-fold and each individual replicate is at least 1.7-fold). (E) Kinetics of binding to RBD variants by Octet of six representative mAbs (representative of n = 2 independent experiments). (F) Distribution of the 144 mAbs based on binding to RBD variants (expressed as fold-change over WT) and hACE2 competition (expressed as the mAb concentration blocking 80% of hACE2 binding, BC80, also indicated as a blue gradient; source data in Data S1). Higher BC80 values (lighter blue) correspond to less hACE2 competition, with mAbs indicated at the top of the panels (white) showing no competition at all. See also Figures S4, S5, and S6.

**Figure S4:**
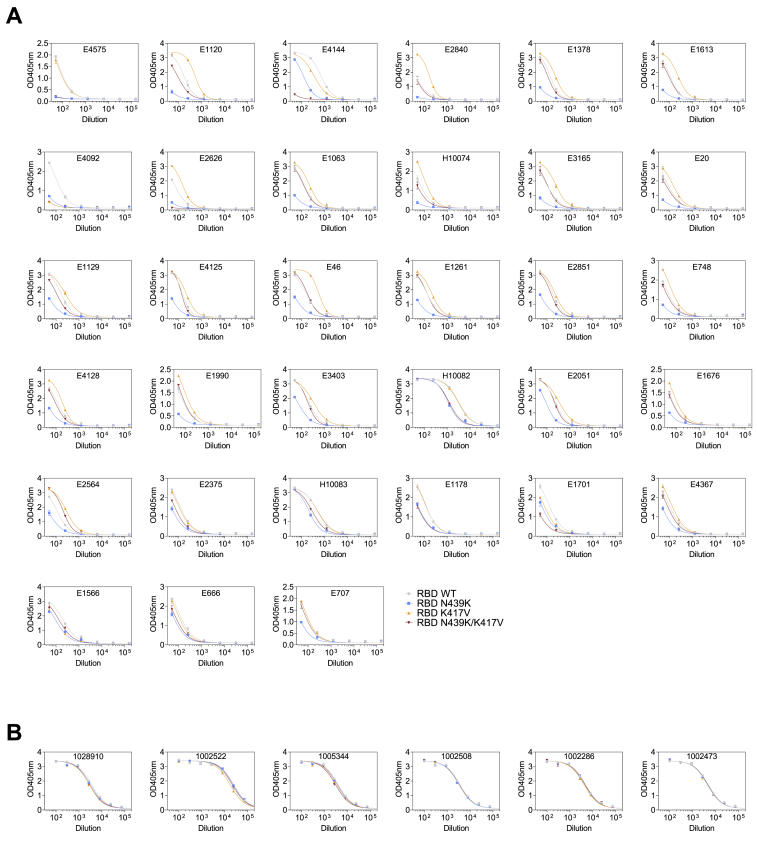
Sera ELISA results, related to Figure 6 ELISA binding of the 33 human sera with a > 2-fold reduction of binding to RBD N439K (A) and of the 6 sera of individuals infected with SARS-CoV-2 N439K viruses (B) to RBD WT (gray), N439K (blue), K417V (yellow) and N439K/K417V (red). Representative of n = 2 independent experiments.

Having established that the N439K mutation has no detectable effect on virus replication, we sought to test whether it promotes evasion of antibody-mediated immunity by evaluating recognition of N439K RBD by mAbs and by polyclonal immune serum from 442 recovered individuals, including six donors who were infected by the SARS-CoV-2 N439K variant. 6.8% of the tested sera showed a >2-fold reduction in binding to N439K RBD as compared to WT (Figures 6A, 6B, and S4; Data S1). In some individuals, the >2-fold reduction diminished the RBD ED_50_ response below 30 (Figure 6A; Data S1), a threshold previously determined to be a cutoff for specific binding (Piccoli et al., 2020). Thus, the response to the RBD can be significantly influenced by the N439K mutation in a number of individuals infected by WT SARS-CoV-2. The majority of serum samples for which there was a loss of binding were those that had overall lower Ab titers against WT RBD. The sera from the six individuals known to have recovered from infection with SARS-CoV-2 N439K virus all showed <2-fold change in binding levels to WT RBD as compared to N439K RBD (Figures 6A, 6B, and S4). This may reflect a true variant-specific response or that differential binding could not be measured due to the limited number of samples analyzed.

**Figure S5:**
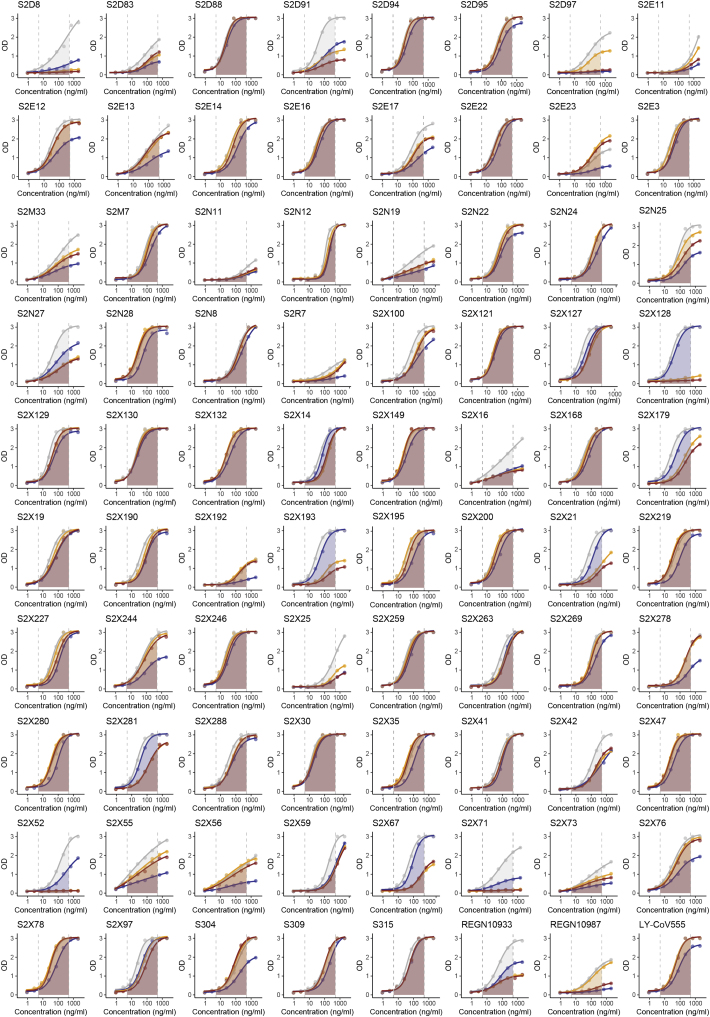
mAb ELISA results, related to Figure 6 ELISA binding of 80 out of the 144 mAbs to RBD WT (gray), N439K (blue), K417V (yellow) and N439K/K417V (red). AUC used for quantification is highlighted between dotted lines. Representative of n = 2 independent experiments. See Data S1 for results of all 144 mAbs.

**Figure S6:**
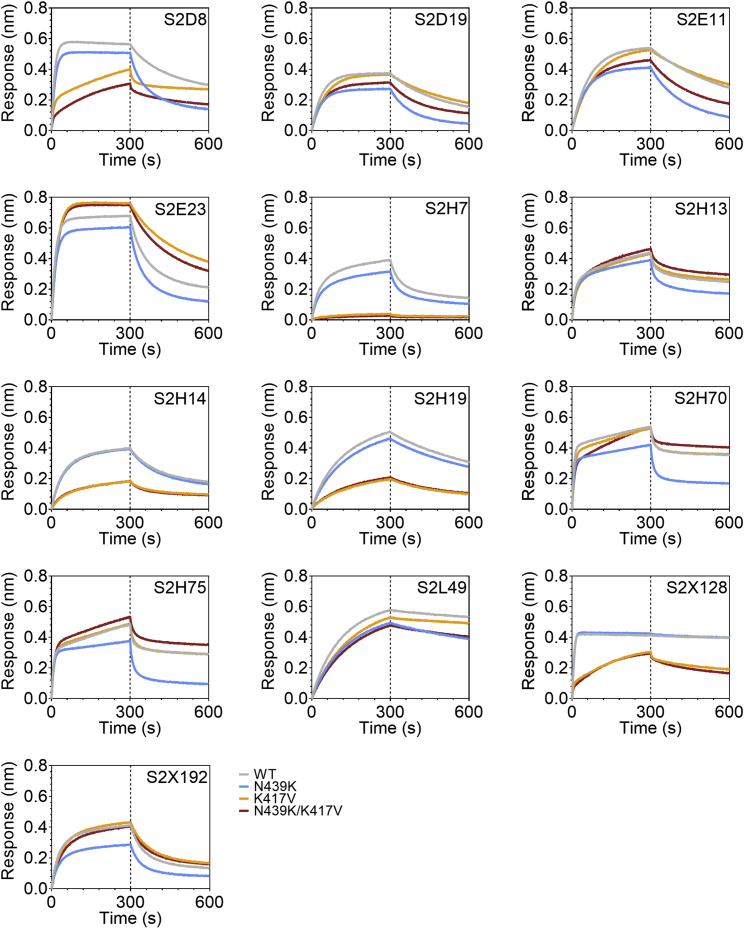
mAb BLI results, related to Figure 6 Binding of 13 selected mAbs to RBD WT (gray), N439K (blue), K417V (yellow) and N439K/K417V (red) as measured by BLI. Representative of n = 2 independent experiments.

To understand our results at the level of individual antibodies, we evaluated a panel of 140 mAbs isolated from individuals recovered from SARS-CoV-2 infection early in the pandemic (likely N439 WT virus), which are a representative sample of the RBD-targeting mAbs generated after infection (Piccoli et al., 2020; Tortorici et al., 2020). We also evaluated mAbs REGN10933, REGN10987, LY-CoV555, and S309 (the parent of VIR-7831) which are either clinical stage or approved for EUA (Baum et al., 2020; Chen et al., 2021; Hansen et al., 2020; Pinto et al., 2020). 16.7% of these mAbs demonstrated a >2-fold reduction of RBD binding in response to the N439K mutation (Figures 6C, 6D, and S5; Data S1). For comparison, we also evaluated the K417V and N439K/K417V mutations. A similar percentage, 9.7% for K417V and 14.6% for N439K/K417V, lost >2-fold binding to these variants (Figures 6C, 6D, and S5; Data S1). Of note, some mAbs demonstrated a larger loss of binding to the double mutant as compared to either single mutant (Figures 6C, 6D; and S5; Data S1). The reduced binding of mAbs to these RBD mutants was also confirmed by bio-layer interferometry analysis (Figures 6E and S6). The mAb panel was evaluated by RBD-binding competition experiments with hACE2 as well as with three structurally characterized antibodies defining distinct epitopes on the RBD: S304/site II, S309/site IV, and S2H14/site I, the latter significantly overlapping with the RBM (Piccoli et al., 2020). The majority of the panel were site I, hACE2-blocking mAbs; the mAbs with sensitivity to N439K were enriched for site I mAbs with moderate or weak/no hACE2 blockade, consistent with the positioning of N439K at the edge of the RBM (Figures 1A and 6F; Data S1).

**Figure 7:**
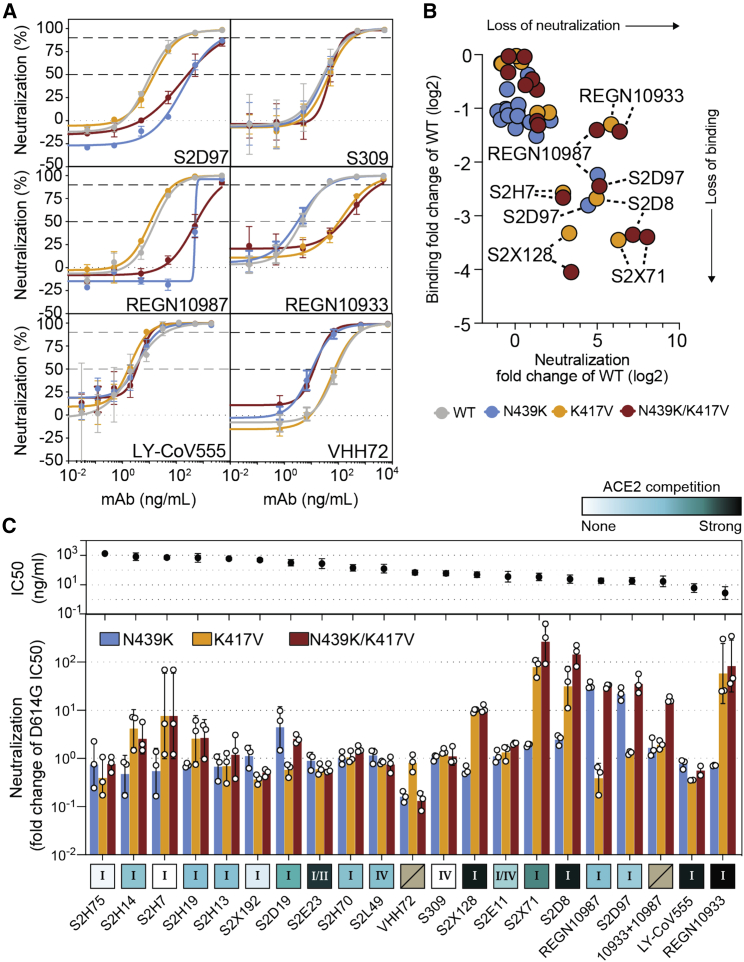
Neutralization of four RBM variants by a panel of antibodies and a nanobody (A) Neutralization of four VSV-pseudovirus variants by six of the mAbs tested. Data shown are representative of n = 3 biological replicates, bars = SD of technical duplicate (Data S1). (B) Correlation of ELISA-binding fold change and neutralization fold change for each variant relative to WT. (C) Top: neutralization IC_50_ of the D614G virus determined as the geometric mean of three biological replicates. Bottom: neutralization results for all mAbs tested, expressed as a fold-change relative to D614G (all variants are in the background of D614G) (Data S1). The individual values of the three replicates are shown as open circles, their geometric mean as colored bars and the geometric SD as error bars. Each antibody is annotated according to its hACE2 competition (as shown in Figure 6F) as well as its epitope (site I, II, or IV) (Data S1). Gray boxes with a slash indicate not tested for hACE2 competition or epitope analysis. See also Figure S7.

**Figure S7:**
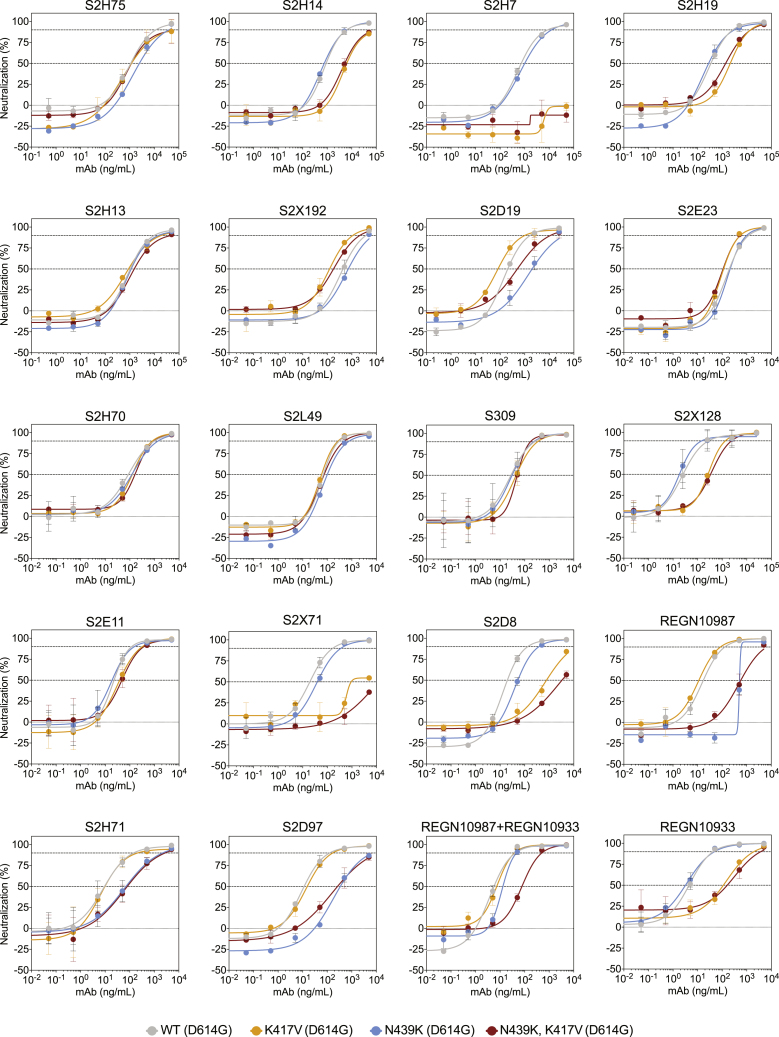
VSV pseudovirus neutralization curves of all mAbs tested, related to Figure 7 Representative of n = 3 biological replicates, bars = SD of technical duplicate.

To define the potential biological importance of these mutations for evasion of antibody-mediated neutralization, we tested mAbs against pseudoviruses expressing S variants N439K, K417V, and N439K/K417V (Figures 7A–7C and S7; Data S1). Neutralization of pseudoviruses containing these mutations was significantly diminished for certain mAbs, including some that are currently in use in patients under EAU. As predicted by its non-RBM epitope (Pinto et al., 2020), S309 was capable of neutralizing each of these variants. We also evaluated a cross-reactive camelid nanobody, VHH-72, which has enhanced potency for SARS-CoV as compared to SARS-CoV-2, predicted to be partially due to a contact with R426 in SARS-CoV RBD, the same position as 439 in SARS-CoV-2 RBD (Wrapp et al., 2020a). Consistent with this prediction, VHH-72 showed enhanced potency against N439K SARS-CoV-2 pseudovirus compared to WT N439 (Figures 7A and 7C), highlighting the possibility that a single mutation can impact antibody efficacy positively as well as negatively. Sensitivity of a few neutralizing mAbs to mutations at positions 417 and 439 have also been reported in other studies (Baum et al., 2020; Gaebler et al., 2021; Greaney et al., 2021; Li et al., 2020a; Starr et al., 2020a; Weisblum et al., 2020), although combinations of mutations have typically not been evaluated. Overall, our results demonstrate that mutations compatible with equivalent viral fitness to WT can result in immune evasion from both monoclonal and polyclonal antibody responses.

## Discussion

Here, we describe an example of a circulating RBM mutation, N439K, which can evade antibody-mediated immunity without losing fitness relative to WT. The success of variants with the N439K mutation is evidenced by their repeated emergence by convergent evolution on at least nine occasions, spread to 34 countries as of January 2021, significant representation in sampled genome sequences (indicative of high infection rates), the fact that the N439K RBD retains a high-affinity interaction with the hACE2 receptor, and efficient replication of N439K virus in cultured cells. Additionally, we observed no evidence for change in disease severity in a large cohort of individuals infected with N439K virus as compared to WT N439 virus, although we acknowledge some limitations in the data collection, including variations in testing guidelines and availability of testing during the course of the study (da Silva Filipe et al., 2021).

The success of the N439K mutation is consistent with our findings that the RBM is a highly variable region of S. It demonstrates the ability of SARS-CoV-2 to accommodate mutations at the RBM while retaining efficient hACE2 binding. This ability could have emerged by chance or in response to immune pressure from neutralizing Ab responses in viral hosts. There is precedent for the most immunogenic region of a viral surface protein to be highly divergent despite harboring the receptor binding site; for example, the immunogenic globular head domain of the influenza virus hemagglutinin surface protein, which contains the sialic acid receptor binding site, evolves faster than the stalk region (Doud et al., 2018; Kirkpatrick et al., 2018). The ability to readily accommodate mutations in the RBM indicates a high likelihood that potentially immune-evading SARS-CoV-2 variants will continue to emerge, with implications for reinfection, vaccines, and both monoclonal and polyclonal antibody therapeutics.

A few other circulating RBM mutations have become prominent since N439K first emerged. S477N appeared in the sequence databases in March 2020 but did not become the most prevalent RBD mutation until the summer (as of January 2021, it has >19,000 counts). Consistent with the high prevalence, position 477 is the RBM position where mutations are predicted to be the most well-tolerated with respect to hACE2 binding (Figure 2). Studies across multiple mAb panels have not found this mutation to be conferring resistance (Gaebler et al., 2021; Greaney et al., 2021; Tortorici et al., 2020; Weisblum et al., 2020). In contrast, mutations at position 484 in the RBM have been reported to confer resistance to many mAbs across multiple studies (Baum et al., 2020; Gaebler et al., 2021; Greaney et al., 2021; Tortorici et al., 2020; Weisblum et al., 2020) and also appeared in a persistent (>150 days) infection in an immunocompromised individual who did not respond to treatment with a two-mAb cocktail (Choi et al., 2020). The variant count at position 484 has been steadily increasing (>500 as of January 2021), and the possibility for no fitness consequences for these variants is notable (Figures 2 and S1B) (Starr et al., 2020b), as is a recent study identifying this position as particularly important for escape from polyclonal serum antibodies (Greaney et al., 2021a) and the appearance of this mutation in a new, fast-growing viral lineage (Tegally et al., 2020). The Y453F mutation has become noteworthy recently for its association with virus circulating in mink farms and its transmission back to humans (Oude Munnink et al., 2021) and the DMS measurement indicating it confers significantly increased hACE2 binding (Starr et al., 2020b). To date, we know of only one example of published immune escape documented for Y453F (Baum et al., 2020), but more examples may arise as this new mutation is investigated further. Last, the N501Y mutation has gained notice in the final weeks of 2020 for its association with high rates of infection (Tegally et al., 2020; Volz et al., 2021), although further research is needed to determine the impact on immune escape.

SARS-CoV-2 appears to be evolving relatively slowly consistent with its low mutation rate, the highly susceptible human population, and its generalist nature (Conceicao et al., 2020) and, at present, evidence indicates it will be controllable by vaccines based on early SARS-CoV-2 genome sequences (Dearlove et al., 2020). Additionally, for the majority of our tested sera and mAbs, a single amino acid change in the RBM was not sufficient to confer resistance. Nevertheless, our data indicate that individuals with a mild antibody response to vaccination or first infection could be at risk from a virus carrying a mutation in the RBM. Furthermore, considering the high level of structural plasticity of the RBM demonstrated in the present study, there could be many combinations of RBM mutations, including some requiring compensatory changes, that are compatible with high viral fitness. Some of these combinations will contribute to efficient immune escape. For example, our data show that N439K can compensate for a mutation (K417V) that otherwise decreases receptor binding affinity (Figure 4D) and that several mAbs were more sensitive to these mutations in combination versus individually (Figure 6D; Data S1). This particular combination of mutations is plausibly compatible with maintained viral fitness as it parallels SARS-CoV RBM:hACE2 interactions (salt bridge at SARS-CoV RBD position R426 and no salt bridge at V404) (Figure 4A). Current SARS-CoV-2 mutations have arisen in the absence of pressure from significant population immunity. However, as immunity to the WT virus becomes more widespread, immune escape mutations can be expected to increasingly circulate. In the final weeks of 2020, SARS-CoV-2 variants carrying multiple mutations in the S protein, in both the RBM and Domain A, have been observed (Volz et al., 2021) including one variant carrying three simultaneous RBM mutations (K417N, E484K, and N501Y) (Tegally et al., 2020). Accumulation of multiple changes may increase the risk of immune escape from vaccines that are based on early SARS-CoV-2 sequences.

Mutations in the RBM will also impact the prophylactic or therapeutic use of mAbs. In our profile of immune escape from the N439K variant, we observed resistance to a mAb which is part of a two-mAb cocktail that recently received EAU. The promise of using cocktails of mAbs is that they should significantly lower the likelihood of drug-induced selection of resistant viruses (Baum et al., 2020). However, if circulating viral variants already carry resistant mutations to one antibody in the cocktail, this could cause the cocktail to be reduced to a monotherapy. Additionally, we observed that two mutations together (N439K/K417V) conferred resistance *in vitro* to the two-mAb cocktail (Figure 7C).

Two approaches will be critical for minimizing the impact of mAb escape mutations. One is to develop mAbs with epitopes that are highly resistant to viral escape. This may include epitopes outside of the RBM and/or epitopes that are cross-reactive across SARS-CoV and SARS-CoV-2, indicating conserved epitopes with a low tolerance for mutation (Garrett Rappazzo et al., 2021; Pinto et al., 2020; Wec et al., 2020; Wrapp et al., 2020a). A comparison of epitopes of RBM-targeting mAbs with the most conserved regions of the RBM (Figure 2) may also identify RBM mAbs with a higher barrier to escape. The second will be to screen patients, likely at the population level, for the presence of potential resistance variants prior to drug administration. The availability of multiple different mAb therapeutics in the clinic could provide the opportunity to tailor the choice of therapeutics to local circulating variants.

In general, given that access to therapeutic mAbs is expanding, and as more people develop immune responses to the WT virus via infection or vaccination, monitoring the evolution of SARS-CoV-2 for escape mutants will be critical. Although we only report on evasion of antibody-mediated immunity here, it would be surprising if similar changes are not observed that confer evasion of T cell immunity and innate immunity.

### Limitations of study

This study presents the finding that the RBM is a highly variable region of the SARS-CoV-2 S protein, and we provide a thorough characterization of the N439K RBM amino acid replacement, and the ability of this mutation to confer immune evasion without attenuating (or enhancing) fitness or disease. When this study was initiated in June 2020, the general consensus was that the slow rate of evolution of SARS-CoV-2 would result in no immediate threat to vaccines or therapies. N439K was the first RBM amino acid replacement, relative to the ancestral SARS-CoV-2 variant used in vaccine preparations, to increase to high frequency and so can be viewed as a sentinel mutation for SARS-CoV-2 antigenic drift. Since initial submission to Cell in late October 2020, the emergence of multiple highly transmissible variants carrying other RBM mutations of significance has brought the study of immune evasion variants to the forefront of SARS-CoV-2 research. Future retrospective studies will confirm whether these new RBM mutations fall into the same category as N439K: mutations that do not attenuate viral fitness or disease but cause immune evasion. Our results on the plasticity of the RBM also anticipated the emergence of the RBM mutations present in the SARS-CoV-2 variants of concern.

## STAR★methods

### Key resources table

**Table:** 

REAGENT or RESOURCE	SOURCE	IDENTIFIER
**Antibodies**		

S304, S309 IgG and Fab fragments	Pinto et al., 2020	PDB: 7JX3
S2H13, S2H14, S2A4, S2X35 IgG	Piccoli et al., 2020	PDB: 7JV2, 7JXC, 7JXD, 7JXE
S2E12, S2M11 IgG	Tortorici et al., 2020	PDB: 7K3Q, 7K43
LY-CoV555 IgG	Eli Lilly and Company	N/A
REGN10933, REGN10987 IgG	Hansen et al., 2020	PDB: 6XDG
VHH-72	Wrapp et al., 2020a	PDB: 6WAQ
Panel of Human IgG	This study	N/A
Goat Anti-Human IgG-AP	Southern Biotech	Cat. No. 2040-04; RRID:AB_2795643
Goat F(ab’)2 Anti-Mouse IgG(H+L), Human ads-AP	Southern Biotech	Cat. No. 1030-04; RRID:AB_2794293
Anti-Avi-tag Antibody, pAb, Rabbit	GenScript	Cat. No. A00674; RRID:AB_915553

**Bacterial strains and pseudotype viruses**		

VSV-G-glycoprotein-pseudotyped virus	Kerafast	Cat. No. EH1020-PM
VSV-SARS-CoV-2 S-glycoprotein-pseudotyped virus	This study	N/A

**Biological samples**		

Serum and plasma of SARS-CoV-2 infected individuals	Piccoli et al., 2020	N/A
Serum from SARS-CoV-2 N439K infected individuals	ISARIC4C https://isaric4c.net/	N/A
Nasopharyngeal swabs from SARS-CoV-2 infected individuals	West of Scotland Specialist Virology Centre	N/A
Sputum from SARS-CoV-2 infected individuals	West of Scotland Specialist Virology Centre	N/A

**Chemicals and recombinant proteins**		

PEI MAX	Polysciences	Cat. No. POL24765-1
TransIT-Lenti	Mirus	Cat. No. 6600
4-Nitrophenyl phosphate disodium salt hexahydrate (pNPP)	Sigma-Aldrich	Cat. No. N2765-100TAB
Blocker Casein (1%) in PBS	Thermo Fisher Scientific	Cat. No. 37528
Tween 20	Sigma Aldrich	Cat. No. 93773
Bovine Serum Albumine	Sigma	Cat. No. 3059
hACE2, mFc tag	ATUM	N/A
BioLock - Biotin Blocking Solution	IBA GmbH	2-0205-050
PNGase F	New England Biolabs	P0704L
EndoH	New England Biolabs	P0702L
Thrombin	Sigma-Aldrich	T1063-250UN
RBD mouse Fc-tagged	Sino Biological	Cat. No. 40592-V05H
Streptavidin-alkaline phosphatase conjugated	Jackson ImmunoResearch	Cat. No. 016-050-084

**Cell lines**		

ExpiCHO-S	Thermo Fisher Scientific	Cat. No. A29127
Expi293F	Thermo Fisher Scientific	Cat. No. A14527
Lenti-X 293T cells	Takara	Cat. No. 632180
Vero E6 cells	ATCC	Cat. No. CRL-1586

**Commercial assays, kits, and products**		

HiTrap Protein A columns (HiTrap Mab select PrismA)	Cytiva	Cat. No. 17-5498-54P
Strep-Tactin XT Superflow high capacity cartridge	IBA GmbH	Cat. No. 2-4026-001
HisTALON Superflow Cartridges, 5 mL	Takara Bio	Cat. No. 635683
HisTALON Superflow Cartridges, 1 mL	Takara Bio	Cat. No. 635650
Superdex 200 Increase 10/300 GL	Cytiva	Cat. No. 28-9909-44
Superose 6 Increase 10/300 GL	Cytiva	Cat. No. 29-0915-96
StrepTrap HP column, 1 mL	Cytiva	Cat. No. 28-9075-46
ExpiFectamine 293 Transfection Kit	Thermo Fisher Scientific	Cat. No. A14524
ExpiFectamine CHO Transfection Kit	Thermo Fisher Scientific	Cat. No. A29129
ExpiFectamine CHO Transfection Kit	Thermo Fisher Scientific	Cat. No. A29130
HiPrep 26/10 desalting columns	Cytiva	Cat. No. 17-5087-02
CaptureSelect C-tag Affinity Matrix	Thermo Fisher Scientific	Cat. No. 2943072010
Zeba Spin Desalting columns, 7 K MWCO, 5 ml	Thermo Fisher Scientific	Cat. No. 89892
Bio-Glo	Promega	Cat. No. G7940
Biosensor Protein A	FortéBio	Cat. No. 733-2137
MiSeq Reagent v2 500 cycle kit	Illumina	Cat. No. MS-102-2003
NextSeq 500/550 High Output Kit v2.5 (300 Cycles)	Illumina	Cat. No. 20024908
DMEM GlutaMAX	Thermo Fisher Scientific	Cat. No. 10566016
Fetal Bovine Serum (FBS)	Thermo Fisher Scientific	Cat. No. A4766801
DNaseI	Thermo Fisher Scientific	Cat. No. AM2222
Agencourt RNA Clean AMPure XP Beads	Beckman Coulter	Cat. No. A63987
Qubit dsDNA HS Kit	Thermo Fisher Scientific	Cat No. Q32854
SuperScript III	Thermo Fisher Scientific	Cat No. 18080044
NEBNext Ultra II Non-Directional RNA Second Strand Synthesis Module	New England Biolabs	Cat. No. E6111L
Kapa HyperPrep kit	Roche	Cat. No. KK8504
Kapa LTP Library Preparation Kit for Illumina Platforms	Roche	Cat. No. KK8232
NEBNext Multiplex Oligos for Illumina 96 Unique Dual Index Primer Pairs	New England Biolabs	Cat. No. E6442S
High Sensitivity D5000 Screentape	Agilent	Cat. No. 5067-5592
NEB Luna Universal Probe One-Step RT-qPCR Kit	New England Biolabs	Cat No. E3006E
2019-nCoV_N1 assay RT-qPCR assay	Integrated DNA Technologies	Cat No. 10006713
HBS-N buffer	Cytiva	Cat. No. BR100369
HBS-EP+ buffer	Cytiva	Cat. No. BR100669
Series S Sensor Chip CM5	Cytiva	Cat. No. BR100530
Series S Sensor Chip C1	Cytiva	Cat. No. BR100535
Mouse antibody capture kit	Cytiva	Cat. No. BR100838
Twin-Strep-Tag Capture Kit	IBA GmbH	Cat. No. 2-4370-000
EZ-Link NHS-PEG Solid-Phase Biotinylation Kit - Mini-Spin Columns	Thermo Fisher Scientific	Cat. No. 21450
Spectraplate-384 with high protein binding	Perkin Elmer	Cat. No. CUSG83093
Nunc-Immuno plates - 96-well plate, MaxiSorp	Sigma-Aldrich Chemie GmbH	Cat. No. M9410-1CS

**Deposited data**		

SARS-CoV-2 RBD N439K/ hACE2/ S304 Fab/ S309 Fab X-ray structure	This study	PDB: 7L0N
SARS-CoV-2 RBD/hACE2 Molecular Dynamics trajectories	This study	https://covid.molssi.org//simulations/#foldinghome-simulations-of-the-sars-cov-2-spike-rbd-bound-to-human-ace2

**Recombinant DNA**		

Human antibody expression vectors (IgG1)	This study	N/A
Plasmids encoding SARS-CoV-2 2P S ectodomain variants (amino acids 14-1211) with C-terminal AviTag-8xHis-C-tag	This study (ATUM Bio)	GenBank: NC_045512.2
Plasmids encoding SARS-CoV-2 RBD WT or variants (amino acids 328-531) with C-terminal thrombin cleavage site-TwinStrep-8xHis-tag	This study	GenBank: NC_045512.2
Plasmid encoding SARS-CoV-2 RBD WT (amino acids 328-531) with N-terminal ‘ETGT’ and C-terminal GS linker-Strep-8xHis-tag	Piccoli et al., 2020	GenBank: NC_045512.2
Plasmid encoding SARS-CoV-2 RBD N439K (amino acids 328-531) with N-terminal ‘ETGT’ and C-terminal 8xHis-tag	This study	GenBank: NC_045512.2
Plasmids encoding Sarbecovirus RBDs with C-terminal thrombin cleavage site-TwinStrep-8xHis-tag	This study	See Data S1
Plasmid encoding human ACE2 receptor (amino acids 19-615) plus C-terminal thrombin cleavage site-TwinStrep-10xHis-GGG-tag	Piccoli et al., 2020	UniProt: Q9BYF1
Plasmids encoding SARS-CoV-2 D19 Spike variants	This study	N/A

**Software and algorithms**		

Prism	GraphPad	Version 8
UNICORN	Cytiva	Versions 7.3 and 7.5
Biacore T200 Evaluation Software	Cytiva	Version 3.1
SDS software	Thermo Fisher Scientific	Version 2.3
Refmac5	Murshudov et al., 2011	Version 5.8.0258
Coot	Emsley et al., 2010	Version 0.9
XDS	Kabsch, 2010	N/A
MOE	Chemical Computing Group	Version 2019.0102
BioEdit	Tom Hall http://bioedit.software.informer.com/7.0	Version 7.0.5.3
R: A Language and Environment for Statistical Computing	R Foundation for Statistical Computing	Version 4.0.3
Skygrowth	https://github.com/mrc-ide/skygrowth	N/A
SPIn	Liu et al., 2015	Version 1.1
IQ-TREE 2	Minh et al., 2020	Version 2.0.6
lubridate	https://github.com/tidyverse/lubridate	Version 1.7.4
ape	Paradis and Schliep, 2019	Version 5.3
brms	Bürkner, 2018	Version 2.13.5
drc	https://cran.r-project.org/web/packages/drc/drc.pdf	Version 3.0-1
entropy	https://cran.r-project.org/web/packages/entropy/	Version 1.2.1
RcppRoll	https://cran.r-project.org/web/packages/RcppRoll/index.html	Version 0.3.0
MinKNOW	Oxford Nanopore technologies	Version 19.12.6
Porechop	https://github.com/rrwick/Porechop	Version 0.2.4
Guppy basecaller	Oxford Nanopore technologies	Version 3.2.10
Nanopolish	https://github.com/jts/nanopolish	Version 0.11.3
trim_galore	http://www.bioinformatics.babraham.ac.uk/projects/trim_galore/	Version 0.6.5
BWA	Li, 2013	Version 0.7.5
iVar	Grubaugh et al., 2019	Version 1.2.2
Minimap2	Li, 2018	Version 2.17
Baltic Python library	https://github.com/evogytis/baltic	N/A
Artic sequencing bioinformatic pipeline	Artic network https://artic.network/ncov-2019	N/A
Miniconda	Anaconda http://www.anaconda.com	Anaconda Version 2-2.4.0 Miniconda Version 4.9.0
Folding@home	Shirts and Pande, 2000; Zimmerman et al., 2020	N/A
IPython	Perez and Granger, 2007	Version 7.14.0
Jupyter Notebook	Kluyver et al., 2016	Version 6.1.5
MDAnalysis	Michaud-Agrawal et al., 2011; Gowers et al., 2016	Version 1.0.0
NumPy	https://numpy.org	Version 1.19.1
OpenMM	Eastman et al., 2017	Version 7.4.2
OpenMMTools	https://github.com/choderalab/openmmtools	Version 0.20.0
PyMOL	Schrödinger	Version 2.3.2
ISOLDE	Croll, 2018	Version 1.0.1
ChimeraX	Pettersen et al., 2021	Version 1.0
AmberTools	Case et al., 2017	Version 17.0
pdb-tools	Rodrigues et al., 2018	Version 2.0.5
MDTraj	McGibbon et al., 2015	Version 1.9.4
Pandas	https://conference.scipy.org/proceedings/scipy2010/pdfs/mckinney.pdf	Version 1.0.5
Custom code, molecular dynamics set up and processing	This paper	https://github.com/choderalab/rbd-ace2-contact-analysis
Custom code, evaluation of clinical samples	This paper	https://github.com/dpascall/SARS-CoV-2-mutation-analysis

**Instruments**		

ÄKTA Xpress FPLC	Cytiva	N/A
ÄKTA Pure 25	Cytiva	N/A
Synergy H1 Hybrid Multi-Mode plate reader	Biotek	N/A
EL406 washer/dispenser BSL2 M	Biotek	N/A
Biacore T200	Cytiva	N/A
Octet Red96	Pall FortéBio	N/A
7500 Fast Real-Time PCR System	Applied Biosystems	N/A
Illumina MiSeq	Illumina	SY-410-1003
Illumina’sNextSeq550	Illumina	SY-415-1002
Flowcell R9.4.1	Oxford Nanopore technologies	FLO-MIN106D
Envision multimode plate reader	PerkinElmer	2105

### Resource availability

#### Lead contact

Further information and requests for resources and reagents should be directed to the Lead Contact, Gyorgy Snell (gsnell@vir.bio).

#### Materials availability

Materials generated in this study will be made available on request, but we may require a completed materials transfer agreement.

#### Data and code availability

Datasets generated during this study are included in the article or are available from the corresponding authors on request. The X-ray structure data and model has been deposited with accession code PDB: 7L0N. The code used to set up, run, and analyze the molecular dynamics simulations is available at: https://github.com/choderalab/rbd-ace2-contact-analysis. Raw and processed molecular dynamics trajectory data are available at the MolSSI COVID-19 Molecular Structure and Therapeutics Hub: https://covid.molssi.org//simulations/#foldinghome-simulations-of-the-sars-cov-2-spike-rbd-bound-to-human-ace2. Code for evaluation of clinical samples is available from GitHub: https://github.com/dpascall/SARS-CoV-2-mutation-analysis.

### Experimental model and subject details

#### Cell lines

Cell lines were obtained from ATCC (Vero E6) or Thermo Fisher Scientific (Expi293F, ExpiCHO-S). Expi293F and ExpiCHO-S cells were maintained in Expi293 Expression Medium and ExpiCHO- Expression Medium (Thermo Fisher Scientific), respectively.

#### Sample donors

Samples from 442 SARS-CoV-2 infected individuals were obtained from the Ticino healthcare workers cohort (Switzerland), described previously (Piccoli et al., 2020), and under study protocols approved by the local Institutional Review Board (Canton Ticino Ethics Committee, Switzerland). All donors provided written informed consent for the use of blood and blood components (such as PBMCs, sera or plasma). In the Ticino region of Switzerland and during the time period of collection (February-March 2020) no N439K SARS-CoV-2 isolates were reported.

Samples from six N439K variant infected individuals were obtained from the ISARIC4C consortium (https://isaric4c.net/). Ethical approval was given by the South Central-Oxford C Research Ethics Committee in England (reference 13/SC/0149), and by the Scotland A Research Ethics Committee (reference 20/SS/0028). The study was registered at https://www.isrctn.com/ISRCTN66726260.

Residual nucleic acid extracts derived from the nose-throat swabs of 1918 SARS-CoV-2 positive individuals whose diagnostic samples were submitted to the West of Scotland specialist virology center between 3^rd^ March and 30^th^ June 2020 were sequenced as part of the COG-UK consortium under study protocols approved by the relevant national biorepositories (16/WS/0207NHS and 10/S1402/33) (consortiumcontact@cogconsortium.uk, 2020).

### Method details

#### Structural analysis

RBM residues were determined based on the RBD:hACE2 complex crystal structures 2AJF for SARS-CoV (Li et al., 2005) and 6M0J for SARS-CoV-2 (Lan et al., 2020). The 2AJF structure was obtained from the PDB-REDO server (https://pdb-redo.eu) and was subsequently prepared in the molecular modeling software MOE (v2019.0102, https://www.chemcomp.com) using the structure preparation, protonation and energy minimization steps with default settings. RBD residues within 6.0 Å distance of any hACE2 atoms (determined using MOE) were determined for each of the two copies of the complex in the asymmetric unit, and then were combined to obtain the definition of the RBM used in this work (Figure 2). 6M0J was obtained from the Coronavirus Structural Task Force server (https://github.com/thorn-lab/coronavirus_structural_task_force) and was further refined (using Refmac5 v5.8.0258), manually fitted (using Coot v0.9) and prepared (using MOE, as described above) in multiple iterative cycles. The final structure was analyzed for RBD-hACE2 contact residues with a 6.0 Å cutoff to obtain the RBM (using MOE). The final list of RBM residues (Figure 1C) was arrived at by combining the SARS-CoV and SARS-CoV-2 results.

Using MOE, the pairwise binding energy (the sum of van der Waals, ionic, and hydrogen-bond interactions) between each residue in SARS-CoV-2 RBD and each residue in hACE2, and the total binding energy for all interactions, was determined at cutoff distances 3.0 Å, 3.5 Å, 4.0 Å, 4.5 Å, 5.0 Å, 5.5 Å, 6.0 Å, 6.5 Å and 7.0 Å. The percentage of the total binding energy for each interacting RBD residue was calculated for each distance cutoff and was then averaged over all cutoffs. The resulting values are shown in green in Figure 1C.

#### RBM variability across SARS-CoV-2 sequences

Using CoV-GLUE-reported variants (http://cov-glue.cvr.gla.ac.uk/, downloaded from GISAID on November 30^th^ 2020, n = 209,239) the Shannon’s entropy (natural log units, as implemented in the R package entropy) was computed at each residue of the mature (excluding signal peptide) spike protein. Then, entropy was aggregated by domain, in sliding windows (using the R package RcppRoll), or in bins of randomly sampled residues, as detailed in figure legends. Due to the non-normal distribution of variant frequencies, the median rather than the mean was used as the aggregation metric.

As an alternative to entropy, we also quantitated variability by counting the number of variants passing an increasing threshold of supporting sequences. Unlike entropy, this metric only uses variant frequency for thresholding a digital (presence/absence) variant call, hence it is less affected by sampling/deposition bias.

#### Evaluation of deep mutational scanning (DMS) data

The DMS dataset was retrieved from Starr et al. (2020b). Variant-level DMS scores were aggregated by residue by taking the minimum (most disruptive variant) or the average score across all variants of that residue, except for the reference amino acid and stop codons. Alternatively, minimum and average scores were computed only across variants that have been observed as naturally occurring. Data were represented as a heatmap annotated with: frequency of variants from CoV-GLUE (at least 1 supporting sequence per 25,000 deposited sequences was required to call a variant); number of countries in which a variant was observed; and percentage of total binding energy computed from an X-ray crystal structure (*cf.* structural analysis methods section).

#### Molecular dynamics simulations

##### Structure preparation

The RBD:hACE2 complex was constructed from individual RBD (PDB: 6m0j, Chain E) and hACE2 (PDB: 1r42, Chain A) monomers aligned to the full RBD:hACE2 structure (PDB: 6m0j). The 1r42 structure was used for hACE2 because 1) 1r42 is higher resolution (2.20 Å, whereas 6m0j is 2.45 Å) and 2) the electron density map of 1r42 clearly reveals N-acetylglucosamine (NAG) orientation at each glycosylated asparagine residue, providing a reliable building block on which to construct more complex glycan structures. These complex glycans were constructed at each NAG due to earlier work suggesting their role in mediating RBD:hACE2 binding (Zhao et al., 2020), as it is in the spike proteins’ intrinsic RBD dynamics (Casalino et al., 2020).

In order to start from the most reliable structural models, we obtained 6m0j and 1r42 from the Coronavirus Structural Taskforce (CST) database, which contains refined structural models based on careful examination of the electron density. In the RBD of the refined 6m0j structure, amino acid rotamers and peptide bonds were flipped to increase Ramachandran favorability, decrease rotamer outliers, reduce clashes, and improve fit to density. A more detailed summary of the 6m0j refinement details is available at: https://github.com/thorn-lab/coronavirus_structural_task_force/blob/master/pdb/surface_glycoprotein/SARS-CoV-2/6m0j/isolde/notes.txt. The 1r42 refined structure differs from the PDB-deposited structure in that it includes the missing C-terminal domain of hACE2 (copied from the 6m17 PDB structure). A more detailed summary of the 1r42 refinement details is available at: https://github.com/thorn-lab/coronavirus_structural_task_force/blob/master/pdb/human_interaction_partners/ACE2/1r42/isolde/notes.txt.

The resulting RBD and hACE2 monomers were then aligned in PyMOL 2.3.2 (Schrödinger, LLC) to the CST 6m0j structure to create an initial RBD:hACE2 complex. The overall root-mean-square deviation (RMSD) was 0.426 Å and the interface RMSD was 0.405 Å, where RMSD was computed for all atoms and the interface residues were defined as all residues within 4 Å of the other binding partner.

Next, the full glycosylation patterns for hACE2 and RBD glycans were determined from Shajahan et al. (2020) and Watanabe et al. (2020). For the constructed RBD:hACE2 complex, these included sites: N53, N90, N103, N322, N432, N546, and N690 on hACE2 and N343 on the RBD. The glycan structures used for each site (FA2, FA26G1, FA2, FA2, FA2G2, A2, FA2, FA2G2, respectively) correspond to the most stable conformers obtained from multi microsecond MD simulations of cumulative sampling (Harbison et al., 2019). Base NAG residues at the reducing end of each glycan structure were aligned to the corresponding NAG stub in the RBD:hACE2 model in PyMOL 2.3.2 (Schrödinger, LLC) and any resulting clashes were refined in ISOLDE (Croll, 2018). Full details of the glycosylation patterns / structures used and full workflow are available at: https://github.com/choderalab/rbd-ace2-contact-analysis.

#### System solvation and parametrization

The refined glycosylated RBD:hACE2 complex was prepared for simulation using the AmberTools17 tleap suite (Case et al., 2017). All relevant disulfide bridges were specified as well as covalent connectivity within each glycan structure. The glycosylated protein was parameterized with the Amber ff14SB (Maier et al., 2015) and GLYCAM_06j-1 (Kirschner et al., 2008) force fields. The system was solvated using the TIP3P rigid water model (Jorgensen et al., 1983) in a cubic box with 1.5 nm solvent padding on all sides. The solvated system was then minimally neutralized with 0.15 M NaCl using the Li/Merz ion parameters of monovalent ions for the TIP3P water model (12-6 normal usage set) (Li et al., 2015). Full details and tleap scripts can be found at: https://github.com/choderalab/rbd-ace2-contact-analysis.

#### System equilibration

The system was energy-minimized with an energy tolerance of 10 kJ mol^−1^and equilibrated using the OpenMM 7.4.2 (Eastman et al., 2017) Langevin integrator for 300 ns in the NPT (p = 1 atm, T = 310 K) ensemble with a timestep of 4.0 femtoseconds, a collision rate of 1.0 picoseconds ^-1^, and a constraint tolerance of 1 × 10^−5^. Hydrogen atom masses were set to 4.0 amu by transferring mass from connected heavy atoms, bonds to hydrogen were constrained, and center of mass motion was not removed. Pressure was controlled by a molecular-scaling Monte Carlo barostat with an update interval of 25 steps. Non-bonded interactions were treated with the Particle Mesh Ewald method (Darden et al., 1993) using a real-space cutoff of 1.0 nm and the OpenMM (Eastman et al., 2017) default relative error tolerance of 0.0005, with grid spacing selected automatically. For improved stability, the structure was then equilibrated using the OpenMMTools 0.20.0 BAOAB Langevin integrator (Leimkuhler and Matthews, 2013) for 10 ns using all of the same simulation parameters described above. This simulation was subsequently packaged to seed for production simulation on Folding@home (Shirts and Pande, 2000; Zimmerman et al., 2020). Default parameters were used unless noted otherwise. Further details of the equilibration protocol are available at: https://github.com/choderalab/rbd-ace2-contact-analysis

#### Folding@home simulations

The equilibrated structure was then used to initiate parallel distributed MD simulations on Folding@home (Shirts and Pande, 2000; Zimmerman et al., 2020). Simulations were run with OpenMM 7.4.2 (Eastman et al., 2017), Folding@home core22 0.0.13). Production simulations used the same Langevin integrator as the NPT equilibration described above. In total, 2000 independent MD simulations were generated on Folding@home. Conformational snapshots (frames) were stored at an interval of 0.5 ns/frame for subsequent analysis. The resulting final dataset contained 2000 trajectories, 183.8 μs of aggregate simulation time, and 367610 frames. This amount of simulation time corresponds to approximately 13.7 GPU-years on an NVIDIA GeForce GTX 1080Ti. This trajectory dataset with solvent is available at the MolSSI COVID-19 Molecular Structure and Therapeutics Hub: https://covid.molssi.org//simulations/#foldinghome-simulations-of-the-sars-cov-2-spike-rbd-bound-to-human-ace2.

#### Simulation analysis

The longest 1000 trajectories were chosen for analysis, ranging from 90 ns to 230 ns in length, which represent an aggregate simulation time of 118.7 μs. Each frame in a trajectory was aligned using MDAnalysis (Gowers et al., 2016; Michaud-Agrawal et al., 2011) to the equilibrated structure. This was to ensure no crossing of periodic boundary conditions during calculation of distances between residue pairs. The distances between residue pairs (K417-D30, E484-K31, Q493-E35, Q493-K31, G496-K353, G502-K353, Y449-D38, Y449-Q42, K31-E35) were calculated once every 5 frames (2.5 ns) using MDAnalysis after discarding the first 100 frames (50 ns) of each trajectory to ensure relaxation away from the initial seed conformation. Distance was defined as the minimum distance between sidechain heavy atoms for a given residue pair. Further details of the analysis pipeline are available at: https://github.com/choderalab/rbd-ace2-contact-analysis

#### RBM variability across Sarbecoviruses

A pairwise comparison of *Sarbecovirus* RBD sequences (see Table S1) to SARS-CoV-2 RBD was performed by calculating percent identity over a window size of 30 amino acids at each RBD position. For the site-specific entropy plot across the RBD alignment of SARS-CoV-2 and 68 related viruses, entropy for each position *l* (H(*l*)) was calculated using Shannon’s entropy formula with a natural log as implemented in Bioedit (H(*l*) = -Σf(*a,l*)ln(f(*a,l*)); f(*a,l*) being the frequency of amino acid *a* at position *l*).

#### Recombinant glycoprotein production

Prefusion-stabilized SARS-CoV-2 spike protein variants (residues 14-1211), containing the 2P and Furin cleavage site mutations (Walls et al., 2020) with a mu-phosphatase signal peptide and a C-terminal Avi-8xHis-C-tag (ATUM Bio) were expressed in Expi293F cells at 37°C and 8% CO_2_. Transfections were performed using the ExpiFectamine 293 Transfection Kit (Thermo Fisher Scientific). Cell culture supernatant was collected after four days and purified over a 5 mL C-tag affinity matrix (Thermo Fisher Scientific). Elution fractions were concentrated and injected on a Superose 6 Increase 10/300 GL column (Cytiva) with 1x PBS pH 7.4 as running buffer.

SARS-CoV-2 RBD WT (with N-terminal signal peptide and C-terminal thrombin cleavage site-TwinStrep-8xHis-tag) and variants were expressed in Expi293F cells at 37°C and 8% CO_2_. Transfections were performed using the ExpiFectamine 293 Transfection Kit (Thermo Fisher Scientific). Cell culture supernatant was collected three days after transfection and supplemented with 10x PBS to a final concentration of 2.5x PBS (342.5 mM NaCl, 6.75 mM KCl and 29.75 mM phosphates), or 3.2x for RBD N439R. SARS-CoV-2 RBDs were purified using 1 or 5 mL HisTALON superflow cartridges (Takara Bio) and subsequently buffer exchanged into 1x HBS-N buffer (Cytiva) or PBS using Zeba Spin Desalting or HiPrep 26/10 desalting columns.

RBDs from other sarbecoviruses and SARS-CoV-2 RBD WT (with N-terminal signal peptide and ‘ETGT’, and C-terminal GS linker-Strep-8xHis-tag) were expressed in Expi293F cells at 37°C and 8% CO_2_. Cells were transfected using PEI MAX (Polysciences) at a DNA:PEI ratio of 1:3.75. Transfected cells were supplemented three days after transfection with 3 g/L glucose (Bioconcept) and 5 g/L soy hydrolysate (Sigma-Aldrich Chemie GmbH). Cell culture supernatant (423 mL) was collected seven days after transfection and supplemented with 47 mL 10x binding buffer (1 M Tris-HCl, 1.5 M NaCl, 20 mM EDTA, pH 8.0) and 25 mL BioLock (IBA GmbH) and incubated on ice for 30 min. Proteins were purified using a 5 mL Strep-Tactin XT Superflow high capacity cartridge (IBA GmbH) followed by buffer exchange to PBS using HiPrep 26/10 desalting columns (Cytiva).

For S binding measurements, recombinant ACE2 (residues 19-615 from Uniprot Q9BYF1 with a C-terminal thrombin cleavage site-TwinStrep-10xHis-GGG-tag, and N-terminal signal peptide) was expressed in Expi293F cells at 37°C and 8% CO_2_. Transfections were performed using the ExpiFectamine 293 Transfection Kit (Thermo Fisher Scientific). Cell culture supernatant was collected seven or eight days after transfection and supplemented to a final concentration of 80 mM Tris-HCl pH 8.0, 100 mM NaCl, and then incubated with BioLock (IBA GmbH) solution. After filtration through a 0.22 μm filter, ACE2 was purified using a 1 mL StrepTrap HP column (Cytiva) followed by isolation of the monomeric ACE2 by size exclusion chromatography using a Superdex 200 Increase 10/300 GL column (Cytiva) pre-equilibrated in PBS.

For crystallography, the same hACE2 construct as above was expressed in ExpiCHO-S cells at 37°C and 8% CO_2_ with kifunensine added to 10 μM. Transfections were performed using the ExpiFectamine CHO transfection kit (Thermo Fisher Scientific). Cell culture supernatant was collected six days after transfection and supplemented to a final concentration of 80 mM Tris-HCl pH 8.0, 100 mM NaCl, and then incubated with BioLock (IBA GmbH) solution for one hour. hACE2 was purified using a 1 mL StrepTrap HP column (Cytiva).

For SPR binding measurements with surface-captured RBD, recombinant hACE2 (residues 19-615 from Uniprot Q9BYF1 with a C-terminal AviTag-10xHis-GGG-tag, and N-terminal signal peptide) was expressed in HEK293.sus using standard methods (ATUM Bio). Protein was purified via Ni Sepharose resin followed by isolation of the monomeric ACE2 by size exclusion chromatography using a Superdex 200 Increase 10/300 GL column pre-equilibrated with PBS.

For binding measurements with surface-captured hACE2, recombinant hACE2 (residues 18-615 with a C-terminal GS-IgG2a-Mm-Fc tag, and N-terminal signal peptide) was stably transfected in CHO-K1 GS knock-down cell line (ATUM Bio). Protein was purified via protein A and buffer exchanged into PBS.

#### Crystallization, data collection, structure determination, and analysis

The SARS-CoV-2 RBD N439K-hACE2 complex was formed together with two Fab fragments (S304 and S309) to aid in crystallization. Prior to forming the SARS-CoV-2 RBD N439K-ACE2-S304-S309 complex, recombinant hACE2 protein was digested using EndoH (New England Biolabs) and thrombin (Sigma-Aldrich). Recombinant SARS-CoV-2 RBD N439K was digested with PNGase F (New England Biolabs) and thrombin (Sigma-Aldrich). RBD was mixed with a 1.3-fold molar excess of deglycosylated hACE2, S304 Fab, and S309 Fab. The complex was purified on a Superdex 200 10/300 GL column pre-equilibrated with 20 mM Tris-HCl pH 7.5, 150 mM NaCl. Crystals of the SARS-CoV-2 RBD N439K-hACE2-S304-S309 complex were obtained at 20°C by sitting drop vapor diffusion. A total of 200 nL of the complex at 6 mg/mL were mixed with 200 nL mother liquor solution containing 0.1 M ammonium sulfate, 20% v/v ethylene glycol, 10% w/v PEG 8000, and 0.1 M bicine/tris pH 8.5.

Data were collected at the Molecular Biology Consortium beamline 4.2.2 at the Advanced Light Source synchrotron facility in Berkeley, CA. Datasets from two crystals were individually processed and then merged with the XDS software package (Kabsch, 2010) for a final dataset of 2.78 Å in space group P2_1_. The RBD N439K-hACE2-S304-S309 complex structure was solved by molecular replacement using phaser (McCoy et al., 2007) from starting models consisting of RBD-S304-S309 (PDB: 7JX3) and hACE2 (PDB: 6m0j). Several subsequent rounds of model building and refinement were performed using Coot (Emsley et al., 2010), ISOLDE (Croll, 2018), Refmac5 (Murshudov et al., 2011), and MOE (https://www.chemcomp.com), to arrive at a final model for the quarternary complex.

#### Binding measurements using surface plasmon resonance (SPR)

SPR binding measurements were performed using a Biacore T200 instrument. S protein was surface captured via anti-AviTag pAb (Genscript) covalently immobilized on a CM5 chip, RBD protein was surface captured via StrepTactin XT (Twin-Strep-Tag Capture Kit, IBA GmbH) covalently immobilized on a CM5 chip, and ACE2-mFc was surface captured via covalent immobilization of the Cytiva Mouse antibody capture kit on a C1 chip. Running buffer was HBS-EP+ pH 7.4 (Cytiva) and all measurements were performed at 25°C. All experiments were performed as single-cycle kinetics, with a 3-fold dilution series of monomeric hACE2 starting from 300 nM, each concentration injected for 180 s, or a 3-fold dilution series of RBD starting from 50 nM, each concentration injected for 240 s. All data were double reference-subtracted and fit to a binding model using Biacore Evaluation software. For one representative replicate, capture levels were normalized to WT for visualization. Binding data with hACE2 as analyte were fit to a 1:1 binding model. Binding data with RBD as analyte were fit to a Heterogeneous Ligand binding model, due to an artifactual kinetic phase with very slow dissociation that arises when RBD is an analyte; the lower affinity of the two K_D_s reported by the fit is reported as the K_D_ of the RBD-ACE2 interaction (the two reported K_D_s are separated by at least two orders of magnitude for all fits). The measured K_D_ for hACE2 binding to S is likely influenced by conformational dynamics of the RBDs in the context of the prefusion S trimer. Reported K_D_s are an average of 3-4 replicates measured on at least two separate days, with error given as SEM.

Differences between the SPR assay and the published DMS binding assay (Starr et al., 2020b) include using targeted measurements of purified proteins expressed in mammalian cells versus yeast surface display, as well as the use of dimeric hACE2 in the DMS experiment, which incorporates avidity effects into the RBD-hACE2 binding measurements that can mask modest changes in binding affinity.

#### hACE2 binding measurements using bio-layer interferometry (BLI)

Binding measurements of Sarbecovirus RBDs to hACE2 were performed by diluting RBDs to 8 μg/ml in kinetic buffer (PBS supplemented with 0.05% BSA) and immobilization on Anti-Penta-His Biosensors of an Octet RED96 system (FortéBio). RBD-coated biosensors were incubated for 5 min with a solution containing 5, 1 or 0.2 μg/ml of hACE2. A dissociation step was then performed by incubating the biosensors for 10 min in kinetic buffer. The change in material bound to the biosensors caused a shift in the interference pattern that was recorded in real time and plotted using GraphPad Prism 8 software.

#### Epidemiological and genome surveillance

A national sequencing collaboration formed at the start of the epidemic in the UK, CoG-UK consortium (COVID-19 Genomics UK (COG-UK) Consortium, 2020) has facilitated the tracking of SARS-CoV-2 sequences across Scotland since the start of the outbreak in February 2020 and real-time monitoring of genetic changes in the Spike gene that might be associated with changes in virulence or transmissibility. Sequencing was carried out using an amplicon-based protocol in real-time at a rate of up to 300 genomes per week. 50% of samples were selected as surveillance samples, representing Scottish health boards proportionately based on population size, while 50% were selected to allow intervention with local issues such as nosocomial infection in hospitals and nursing homes. The N439K mutation was noted to become increasingly prevalent during April 2020. This was noted to be particularly common in the Greater Glasgow & Clyde NHS health board region but spread to adjacent Scottish health boards also.

Sequencing libraries were prepared according to the ARTIC nCoV-2019 described in detail at https://artic.network/ncov-2019. Briefly, PCR amplicons were generated using the nCoV-2019 PrimalSeq sequencing primers using 25-35 cycles of amplification. Generated amplicons were used to prepare either Oxford Nanopore or Illumina sequencing libraries. Oxford Nanopore libraries were prepared as described in the link above and sequenced in a flow cell R9.4.1 (Oxford Nanopore Technologies, Part Number FLO-MIN106D), using MinKNOW version 19.12.6. Raw FAST5 files were basecalled using Guppy version 3.2.10 in high accuracy mode with a minimum quality score of 7. Reads were size filtered, demultiplexed and trimmed with Porechop (https://github.com/rrwick/Porechop), and mapped against reference strain Wuhan-Hu-1 (MN908947). Variants were called using Nanopolish 0.11.3 and accepted if they had a log-likelihood score of greater than 200 and minimum read coverage of 20. For Illumina sequencing, amplicons were used to prepare libraries using the Kapa HyperPrep kit (Roche, Part Number KK8504) and further processed as described in the competition assay sequencing method. Sequencing was carried out on Illumina’s MiSeq system (Illumina, Part Number SY-410-1003) using a MiSeq Reagent v2 500 cycle kit (Illumina, Part Number MS-102-2003). Reads were trimmed with trim_galore (http://www.bioinformatics.babraham.ac.uk/projects/trim_galore/) and mapped with BWA (Li and Durbin, 2009) to the Wuhan-Hu-1 (MN908947) reference sequence, followed by primer trimming and consensus calling with iVar (Grubaugh et al., 2019) and a minimum read coverage of 10.

#### Phylogenetic and phylodynamic analysis

UK sequences were obtained from the COG-UK consortium (https://www.cogconsortium.uk) and global sequences from the GISAID Initiative (https://www.gisaid.org) on November 23 2020. The sequences were mapped using minimap2 and padded against the Wuhan/WH04/2020 reference. The sequences were downsampled with weights that normalize sequence count per epiweek, maximize the number of countries and lineages represented, and enriching for sequences with the N439K mutation. A maximum-likelihood phylogenetic tree was constructed using IQ-TREE with the the following parameters: -czb -blmin 0.0000000001 -m HKY–runs 5 and all other parameters set to default. The tree was visualized with custom python code using the baltic library (https://github.com/evogytis/baltic).

For the phylodynamic analysis, Scottish “introduction” lineages with ten or more sequences were identified (Lycett et al., 2021), and the skygrowth package in R was used to estimate the effective population size over time (using up to ten time intervals), and the growth rates of the lineages within Scotland (Volz and Frost, 2017). The data used for analysis were sampled between Feb 28, 2020 and Aug 18, 2020. Growth estimates were calculated for the intervals in between the time points that Ne is estimated over, from the TMRCA onward. Lineages with less than ten sequences in total, less than 50% and/or less than five Scottish sequences were excluded.

#### Evaluation of clinical samples

Clinical samples submitted to the West of Scotland Specialist Virology Centre for SARS-CoV-2 diagnostic rt-PCR testing were selected for sequencing as part of the COVID-19 UK Genomics UK Consortium (COG-UK) project, resulting in 1918 whole genome sequences originating from the NHS Greater Glasgow and Clyde Health Board region. Sequences were linked to electronic patient records and basic metadata including sample date, age, sex, admission to hospital and mortality at 28 days post diagnosis extracted. The electronic patient records of a subset of 1591 patients underwent full case-note review and clinical severity was recorded based on a 4-level ordinal scale: 1. no requirement for respiratory support, 2. treatment with supplemental oxygen via facemask or low-flow nasal cannulae, 3. intubation and ventilation, non-invasive ventilation or oxygen delivery by high flow nasal cannulae devices, 4. death within the 28 days following diagnosis. We modified the WHO ordinal scale to these 4 points as described previously (Volz et al., 2021) to avoid using hospitalisation as a criterion of severity because 1) many patients in nursing homes had severe infection but were not admitted to hospital, and 2) early in the outbreak, all cases were hospitalised irrespective of the severity of their infection.

These data had previously been analyzed to test for an effect of the D614G mutation on the severity of disease (Volz et al., 2021); we extend that analysis here using the same methodology to test for an effect of the N439K mutation. Additionally, we perform a new analysis using a model with the same structure to test for an effect of both the D614G mutation and the D614G/N439K mutation combination on the viral load of infected patients, as measured by cycle threshold (Ct) value. Ct values were generated in different locations, on different platforms and then collected centrally.

In both cases we cannot estimate the marginal effect of the N439K mutation, as we only have the mutation on the 614G genetic background, so the individual effect of N439K cannot be separated from any potential epistatic interactions between the mutations.

Briefly, the structure of the model used previously (Volz et al., 2021) and in the present study is a phylogenetic generalized additive model with mutation being the primary predictor of interest. The model controls for biological sex, age and the number of days since the first reported case in the dataset, with the latter two being included as penalised splines with a maximum of 30 knots. If the patient was part of a cluster of cases, this was included as a random effect, with individuals not part of clusters being assigned their own levels. Correlations driven by the rest of the genome are controlled for by a phylogenetic random effect using a correlation matrix generated under a Brownian motion assumption from a phylogeny estimated in IQ-TREE 2 v. 2.0.6 (Minh et al., 2020) using a HKY + Γ model, masking the positions recommended by (De Maio et al. 2020) as of 22/7/2020 (https://virological.org/t/issues-with-sars-cov-2-sequencing-data/473/13), rooted on the first sequenced SARS-CoV-2 genome (Wu et al., 2020a). The priors for the severity model were those used in the previous analysis of this data. The priors for the model of the viral load were a student-t (mean = 20, scale = 10, degrees of freedom = 3) prior on the model intercept, a Gaussian (mean = 0, standard deviation = 10) prior over the fixed effects, and an exponential (lambda = 0.1) prior over the random effect, penalised spline and residual standard deviations.

There are two key structural differences between the model used previously (Volz et al., 2021) and the model used here. First, mutation is a three level rather than two level factor (D614/N439, D614G/N439 and D614G/N439K) with the ancestral D614/N439 being the reference level. Second, as we are now interested in two mutations, we estimated the phylogeny used to control for the effect of the rest of the genome excluding both the nucleotide position underlying the D614G mutation and the nucleotide position underlying the N439K mutation (in addition to the sites from De Maio et al. mentioned above).

The severity model used a cumulative error structure while the model on the CT values used a Gaussian error structure. In both cases, the models were estimated in brms v. 2.13.5 (Bürkner, 2018). The presented models had no divergent transitions, Rhat values less than 1.01, and appropriate bulk and tail effective sample sizes for all parameters. Shortest probability intervals were calculated using the R package SPIn v. 1.1 (Liu et al., 2015). Trees were manipulated using ape v. 5.3 (Paradis and Schliep, 2019), and dates were manipulated using lubridate v. 1.7.4 (Grolemund and Wickham, 2011). Analysis code is available at https://github.com/dpascall/SARS-CoV-2-mutation-analysis.

#### qPCR of clinical samples

All samples were tested in duplicate using the 2019-nCoV_N1 assay RT-qPCR assay (https://www.fda.gov/media/134922/download). Ready-mixed primers and probe were obtained from IDT (Leuven, Belgium). PCR was carried out using NEB Luna Universal Probe One-Step RT-qPCR Kit (New England Biolabs, Herts, UK), primers and probe at 500 nM and 127.5 nM, respectively, and 5 μL of RNA sample in a final volume of 20 μL. No template negative controls were included after every seventh sample. Six ten-fold dilutions of SARS-CoV-2 RNA standards were tested in duplicate in each assay; standards were calibrated using a plasmid containing the N sequence that had been quantified using droplet digital PCR. Thermal cycling was performed on an Applied Biosystems 7500 Fast PCR instrument running SDS software v2.3 (Thermo Fisher Scientific) under the following conditions: 55°C for 10 minutes and 95°C for 1 minute followed by 45 cycles of 95°C for 10 s and 58°C for 1 minute. Assays were repeated if the reaction efficiency was < 90% or the R2 value of the standard curve was ≤ 0.998. Where possible, testing of samples was repeated if the %CV of the duplicates was < 10%.

#### Viral growth curve

Vero E6-hACE2 cells (Vero E6 cells induced to overexpress hACE2) either with or without TMPRSS2 overexpression (S.J.R., S.B., and A.W., unpublished data) were seeded in a 12-well plate and inoculated with an MOI of 0.01 with either the GLA1 (N439/D614G) or GLA2 (N439K/D614G) virus isolates for 1 hr before washing the cells three times in PBS and replacing with DMEM supplemented with 2% FBS. 100 uL of media was removed at each time point, RNA was extracted using the RNAdvance Blood kit (Beckman Coulter), and the presence of SARS-CoV-2 determined using 2019-nCOV-N1 assays (IDT) with an NEB Luna Universal Probe One-Step RT-qPCR Kit. A standard curve was used to determine the copy number present per mL of cell culture media. 100 uL of the fresh media was also tested for the presence of virus, which was undetectable in all wells. Experiment was performed in triplicate, each with an independent preparation of virus inoculum.

#### Competition assay

Three T25 flasks were seeded with Vero E6-hACE2 or Vero E6-hACE2-TMPRSS2 and inoculated with either single viruses or both GLA1 and GLA2 virus strains at an MOI of 0.01 for 1 hr. The flasks were washed three times with PBS, with 100 uL of the final wash being retained to determine the presence of free virus, before adding 5 mL of fresh DMEM, supplemented with 2% FBS. At 24, 48, and 72 hr, 500 uL of media was removed, which was replaced with 500 uL fresh media. 300 uL was used for RNA extraction using the RNAdvance Blood kit (Beckman Coulter) and NGS analysis of the frequencies of the specific positions within the spike protein. The single virus inoculations showed no alternations in the frequency of the amino acid positions and the final wash showing no free virus in the supernatant. We used an unbiased metagenomic NGS sequencing pipeline to quantify variation across the whole viral genome on the Illumina NGS Next Seq platform. Briefly, extracted nucleic acid was incubated with DNaseI (Thermo Fisher Scientific, Part Number AM2222), cleaned with RNA Ampure beads (Agencourt RNA Clean AMPure XP Beads, Beckman Coulter, Cat. No. A63987) followed by cDNA synthesis using SuperScript III (Thermo Fisher Scientific, Part Number 18080044) and NEBNext Ultra II Non-Directional RNA Second Strand Synthesis Module (New England Biolabs, Part Number E6111L). Samples were further processed using the Kapa LTP Library Preparation Kit for Illumina Platforms (Roche, Part Number KK8232) and indexed with the NEBNext Multiplex Oligos for Illumina 96 Unique Dual Index Primer Pairs (New England Biolabs, Part Number E6442S). Libraries were characterized utilizing using the Qubit dsDNA HS Kit (Thermo Fisher Scientific, Cat No. Q32854) and the sequenced on Illumina’s NextSeq 550 System (Illumina, Part Number SY-415-1002), using a NextSeq 500/550 High Output Kit v2.5 (300 Cycles) (Illumina, Cat. No. 20024908) generating aproximately 10 million pairs of reads per sample. Experiment was performed in triplicate, each with an independent preparation of virus inoculum.

#### Ab discovery and recombinant expression

Human mAbs were isolated from plasma cells or memory B cells of SARS-CoV or SARS-CoV-2 immune donors, as previously described (Corti et al., 2011; Pinto et al., 2020; Tortorici et al., 2020). Recombinant antibodies were expressed in ExpiCHO cells at 37°C and 8% CO_2_. Cells were transfected using ExpiFectamine. Transfected cells were supplemented 1 day after transfection with ExpiCHO Feed and ExpiFectamine CHO Enhancer. Cell culture supernatant was collected eight days after transfection and filtered through a 0.2 μm filter. Recombinant antibodies were affinity purified on an ÄKTA xpress FPLC device using 5 mL HiTrap MabSelect PrismA columns followed by buffer exchange to Histidine buffer (20 mM Histidine, 8% sucrose, pH 6) using HiPrep 26/10 desalting columns.

#### Enzyme-linked immunosorbent assay (ELISA)

A total of 144 human monoclonal antibodies or 442 human sera were tested for binding to RBD WT and mutants, as previously described (Piccoli et al., 2020). Spectraplate-384 plates with high protein binding treatment (custom made from Perkin Elmer) were coated overnight at 4 °C with 0.1 μg/mL (for mAbs) or 5 ug/mL (for sera) SARS-CoV-2 RBD WT, N439K, K417V or N439K/K417V in phosphate-buffered saline (PBS), pH 7.2. Plates were subsequently blocked with Blocker Casein 1% supplemented with 0.05% Tween 20 (Sigma-Aldrich) for 1 h at room temperature. The coated plates were incubated with serial dilutions of the monoclonal antibodies or of the sera for 1 h at room temperature. The plates were then washed with PBS containing 0.05% Tween-20 (PBS-T), and alkaline phosphatase-goat anti-human IgG (Southern Biotech) was added and incubated for 1 h at room temperature. After 3 washing steps with PBS-T, p-NitroPhenyl Phosphate (pNPP, Sigma-Aldrich) substrate was added and incubated for 30 min at room temperature. The absorbance of 405 nm was measured by a microplate reader (Synergy H1 Hybrid Multi-Mode plate reader, Biotek). For mAbs, fitting was performed using the drc R package with a 4-parameter logistic (4PL) model, yielding dose-response curves from which the area under the curve (AUC) between 5 and 500 ng/mL was computed. The AUC allows to capture, in a single metric, shifts in two parameters of the 4PL model: EC_50_ and upper asymptote. For sera, fitting was performed using GraphPrism 8 with a 4PL model from which ED_50_ was calculated. A cutoff of 30 for serum ED_50_ was set based on previously published data (Piccoli et al., 2020). A difference in reactivity to RBD mutants compared to WT was considered when a minimum 2-fold-variation in the AUC or ED_50_ WT/mutant ratios was observed in two independent experiments (for mAbs, fold variation as low as 1.7 was accepted provided that the average of two experiments was greater or equal to 2-fold).

#### Antibody binding measurements using bio-layer interferometry (BLI)

BLI binding measurement was performed on a selection of human monoclonal antibodies tested by ELISA (16/144). Antibodies were diluted to 2.7 μg/mL in kinetic buffer (PBS supplemented with 0.05% BSA) and immobilized on Protein A Biosensors of an Octet RED96 system (FortéBio). Antibody-coated biosensors were incubated for 5 min with a solution containing 5 μg /mL of SARS-CoV2 RBD WT, N439K, K417V or N439/K417V in kinetic buffer. A dissociation step was then performed by incubating the biosensors for 5 min in kinetic buffer. Change in molecules bound to the biosensors caused a shift in the interference pattern that was recorded in real time and plotted using GraphPad Prism 8 software.

#### Blockade of RBD binding to ACE2

Blockade of WT RBD binding to hACE2 was performed, as previously described (Piccoli et al., 2020). Unlabeled mAbs were serially diluted, mixed with RBD mouse Fc-tagged antigen (Sino Biological, final concentration 20 ng/mL) and incubated for 30 min at 37°C. The mix was added for 30 min to ELISA 96-well plates (Corning) pre-coated overnight at 4°C with 2 μg/mL hACE2 in PBS. Plates were washed (EL406 washer/dispenser BSL2 M, Biotek) and RBD binding was revealed using a secondary goat anti-mouse IgG (Southern Biotech). After washing, pNPP substrate was added and plates were read at 405 nm (Synergy H1 Hybrid Multi-Mode plate reader, Biotek). The percentage of inhibition was calculated as follow: (1−(OD sample−OD neg ctr)/(OD pos ctr−OD neg ctr)) × 100.

#### RBD epitope mapping (blockade of binding assay)

RBD epitope mapping of the 144 mAbs was performed through blockade of binding (BOB) assay as previously described (Piccoli et al., 2020). Human mAbs binding to RBD site I (S2H14), site II (S304) and site IV (S309) were biotinylated using the EZ-Link NHS-PEO solid phase biotinylation kit (Pierce). Labeled mAbs were tested for binding to RBD by ELISA and the optimal concentration of each mAb to achieve 80% maximal binding was determined. Unlabeled mAbs were serially diluted and added to ELISA 96-well plates (Corning) pre-coated overnight at 4°C with 1 μg/mL of RBD mouse Fc-tagged antigen (Sino Biological) in PBS. After 30 min, biotinylated anti-RBD mAbs were added at the concentration achieving 80% maximal binding and the mixture was incubated at room temperature for 20 min. Plates were washed (EL406 washer/dispenser BSL2 M, Biotek) and antibody binding was revealed using alkaline phosphatase-conjugated streptavidin (Jackson ImmunoResearch). After washing, pNPP substrate (Sigma-Aldrich) was added and plates were read at 405 nm (Synergy H1 Hybrid Multi-Mode plate reader, Biotek). The percentage of inhibition was calculated as follow: (1−(OD sample−OD neg ctr)/ (OD pos ctr−OD neg ctr)) × 100.

#### VSV pseudovirus generation

Replication defective VSV pseudovirus (Takada et al., 1997) expressing SARS-CoV-2 spike protein were generated as previously described (Riblett et al., 2015) with some modifications. Plasmids encoding SARS-CoV-2 spike variants were generated by site-directed mutagenesis of the wild-type plasmid, pcDNA3.1(+)-spike-D19 (Giroglou et al., 2004). Lenti-X 293T cells (Takara, 632180) were seeded in 10-cm dishes at a density of 1e5 cells/cm^2^ and the following day transfected with 5 μg of spike expression plasmid with TransIT-Lenti (Mirus, 6600) according to the manufacturer’s instructions. One day post-transfection, cells were infected with VSV-luc (VSV-G) (Kerafast, EH1020-PM) for 1 h, rinsed three times with PBS, then incubated for an additional 24 h in complete media at 37°C. The cell supernatant was clarified by centrifugation, filtered (0.45 μm), aliquoted, and frozen at −80°C.

#### Pseudovirus neutralization

Vero E6 cells (ATCC CRL-1586) were grown in DMEM supplemented with 10% FBS and seeded into clear bottom white 96 well plates (Costar, 3903) at a density of 2e4 cells per well. The next day, mAbs were serially diluted in pre-warmed complete media, mixed at a 1:1 ratio with pseudovirus and incubated for 1 h at 37°C in round bottom polypropylene plates. Media from cells was aspirated and 50 μL of virus-mAb complexes were added to cells and then incubated for 1 h at 37°C. An additional 100 μL of prewarmed complete media was then added on top of complexes and cells incubated for an additional 16-24 h. Conditions were tested in duplicate wells on each plate and at least six wells per plate contained uninfected, untreated cells (mock) and infected, untreated cells (‘no mAb control’). Virus-mAb-containing media was then aspirated from cells and 100 uL of a 1:4 dilution of Bio-glo (Promega, G7940) in PBS was added to cells. Plates were incubated for 10 min at room temperature and then were analyzed on the Envision plate reader (PerkinElmer). Relative light units (RLUs) for infected wells were subtracted by the average of RLU values for the mock wells (background subtraction) and then normalized to the average of background subtracted “no mAb control” RLU values within each plate. Percent neutralization was calculated by subtracting from 1 the normalized mAb infection condition. Data were analyzed and visualized with Prism (Version 8.4.3). IC_50_ and IC80 values were calculated from the interpolated value from the log(inhibitor) versus response – variable slope (four parameters) nonlinear regression with an upper constraint of < 100. Each neutralization experiment was conducted on three independent days, i.e., biological replicates, where each biological replicate contains a technical duplicate. IC_50_ values across biological replicates are presented as geometric mean ± geometric standard deviation. The loss or gain of neutralization potency across spike variants was calculated by dividing the variant IC_50_ by the parental (D614G) IC_50_ within each biological replicate, and then visualized as geometric mean ± geometric standard deviation.

### Quantification and statistical analysis

Quantification and statistical analyses were performed using GraphPad Prism (v8), R, and Biacore T200 Evaluation software, as described in the Method details.

## Consortia

The members of the COG-UK consortia are Thomas R. Connor, Nicholas J. Loman, Samuel C. Robson, Tanya Golubchik, M. Estee Torok, William L. Hamilton, David Bonsall, Ali R. Awan, Sally Corden, Ian Goodfellow, Darren L. Smith, Martin D. Curran, Surendra Parmar, James G. Shepherd, Matthew D. Parker, Catherine Moore, Derek J. Fairley, Matthew W. Loose, Joanne Watkins, Matthew Bull, Sam Nicholls, David M. Aanensen, Sharon Glaysher, Matthew Bashton, Nicole Pacchiarini, Anthony P. Underwood, Thushan I. de Silva, Dennis Wang, Monique Andersson, Anoop J. Chauhan, Mariateresa de Cesare, Catherine Ludden, Tabitha W. Mahungu, Rebecca Dewar, Martin P. McHugh, Natasha G. Jesudason, Kathy K. Li, Rajiv N. Shah, Yusri Taha, Kate E. Templeton, Simon Cottrell, Justin O’Grady, Andrew Rambaut, Colin P. Smith, Matthew T.G. Holden, Emma C. Thomson, Samuel Moses, Meera Chand, Chrystala Constantinidou, Alistair C. Darby, Julian A. Hiscox, Steve Paterson, Meera Unnikrishnan, Andrew J. Page, Erik M. Volz, Charlotte J. Houldcroft, Aminu S. Jahun, James P. McKenna, Luke W. Meredith, Andrew Nelson, Sarojini Pandey, Gregory R. Young, Anna Price, Sara Rey, Sunando Roy, Ben Temperton, Matthew Wyles, Stefan Rooke, Sharif Shaaban, Helen Adams, Yann Bourgeois, Katie F. Loveson, Áine O’Toole, Richard Stark, Ewan M. Harrison, David Heyburn, Sharon J. Peacock, David Buck, Michaela John, Dorota Jamrozy, Joshua Quick, Rahul Batra, Katherine L. Bellis, Beth Blane, Sophia T. Girgis, Angie Green, Anita Justice, Mark Kristiansen, Rachel J. Williams, Radoslaw Poplawski, Garry P. Scarlett, John A. Todd, Christophe Fraser, Judith Breuer, Sergi Castellano, Stephen L. Michell, Dimitris Gramatopoulos, Jonathan Edgeworth, Gemma L. Kay, Ana da Silva Filipe, Aaron R. Jeffries, Sascha Ott, Oliver Pybus, David L. Robertson, David A. Simpson, Chris Williams, Cressida Auckland, John Boyes, Samir Dervisevic, Sian Ellard, Sonia Goncalves, Emma J. Meader, Peter Muir, Husam Osman, Reenesh Prakash, Venkat Sivaprakasam, Ian B. Vipond, Jane A.H. Masoli, Nabil-Fareed Alikhan, Matthew Carlile, Noel Craine, Sam T. Haldenby, Nadine Holmes, Ronan A. Lyons, Christopher Moore, Malorie Perry, Ben Warne, Thomas Williams, Lisa Berry, Andrew Bosworth, Julianne Rose Brown, Sharon Campbell, Anna Casey, Gemma Clark, Jennifer Collins, Alison Cox, Thomas Davis, Gary Eltringham, Cariad Evans, Clive Graham, Fenella Halstead, Kathryn Ann Harris, Christopher Holmes, Stephanie Hutchings, Miren Iturriza-Gomara, Kate Johnson, Katie Jones, Alexander J. Keeley, Bridget A. Knight, Cherian Koshy, Steven Liggett, Hannah Lowe, Anita O. Lucaci, Jessica Lynch, Patrick C McClure, Nathan Moore, Matilde Mori, David G. Partridge, Pinglawathee Madona, Hannah M. Pymont, Paul Anthony Randell, Mohammad Raza, Felicity Ryan, Robert Shaw, Tim J. Sloan, Emma Swindells, Alexander Adams, Hibo Asad, Alec Birchley, Tony Thomas Brooks, Giselda Bucca, Ethan Butcher, Sarah L. Caddy, Laura G. Caller, Yasmin Chaudhry, Jason Coombes, Michelle Cronin, Patricia L. Dyal, Johnathan M. Evans, Laia Fina, Bree Gatica-Wilcox, Iliana Georgana, Lauren Gilbert, Lee Graham, Danielle C. Groves, Grant Hall, Ember Hilvers, Myra Hosmillo, Hannah Jones, Sophie Jones, Fahad A. Khokhar, Sara Kumziene-Summerhayes, George MacIntyre-Cockett, Rocio T. Martinez Nunez, Caoimhe McKerr, Claire McMurray, Richard Myers, Yasmin Nicole Panchbhaya, Malte L. Pinckert, Amy Plimmer, Joanne Stockton, Sarah Taylor, Alicia Thornton, Amy Trebes, Alexander J. Trotter, Helena Jane Tutill, Charlotte A. Williams, Anna Yakovleva, Wen C. Yew, Mohammad T. Alam, Laura Baxter, Olivia Boyd, Fabricia F. Nascimento, Timothy M. Freeman, Lily Geidelberg, Joseph Hughes, David Jorgensen, Benjamin B. Lindsey, Richard J. Orton, Manon Ragonnet-Cronin, Joel Southgate, Sreenu Vattipally, Igor Starinskij, Joshua B. Singer, Khalil Abudahab, Leonardo de Oliveira Martins, Thanh Le-Viet, Mirko Menegazzo, Ben E.W. Taylor, Corin A. Yeats, Sophie Palmer, Carol M. Churcher, Alisha Davies, Elen De Lacy, Fatima Downing, Sue Edward, Nikki Smith, Frances Bolt, Alex Alderton, Matt Berriman, Ian G. Charles, Nicholas Cortes, Tanya Curran, John Danesh, Sahar Eldirdiri, Ngozi Elumogo, Andrew Hattersley, Alison Holmes, Robin Howe, Rachel Jones, Anita Kenyon, Robert A. Kingsley, Dominic Kwiatkowski, Cordelia Langford, Jenifer Mason, Alison E. Mather, Lizzie Meadows, Sian Morgan, James Price, Trevor I. Robinson, Giri Shankar, John Wain, Mark A. Webber, Declan T. Bradley, Michael R. Chapman, Derrick Crooke, David Eyre, Martyn Guest, Huw Gulliver, Sarah Hoosdally, Christine Kitchen, Ian Merrick, Siddharth Mookerjee, Robert Munn, Timothy Peto, Will Potter, Dheeraj K Sethi, Wendy Smith, Luke B. Snell, Rachael Stanley, Claire Stuart, Elizabeth Wastenge, Erwan Acheson, Safiah Afifi, Elias Allara, Roberto Amato, Adrienn Angyal, Elihu Aranday-Cortes, Cristina Ariani, Jordan Ashworth, Stephen Attwood, Alp Aydin, David J. Baker, Carlos E. Balcazar, Angela Beckett, Robert Beer, Gilberto Betancor, Emma Betteridge, David Bibby, Daniel Bradshaw, Catherine Bresner, Hannah E. Bridgewater, Alice Broos, Rebecca Brown, Paul E. Brown, Kirstyn Brunker, Stephen N. Carmichael, Jeffrey K.J. Cheng, Rachel Colquhoun, Gavin Dabrera, Johnny Debebe, Eleanor Drury, Louis du Plessis, Richard Eccles, Nicholas Ellaby, Audrey Farbos, Ben Farr, Jacqueline Findlay, Chloe L. Fisher, Leysa Marie Forrest, Sarah Francois, Lucy R. Frost, William Fuller, Eileen Gallagher, Michael D. Gallagher, Matthew Gemmell, Rachel A.J. Gilroy, Scott Goodwin, Luke R. Green, Richard Gregory, Natalie Groves, James W. Harrison, Hassan Hartman, Andrew R. Hesketh, Verity Hill, Jonathan Hubb, Margaret Hughes, David K. Jackson, Ben Jackson, Keith James, Natasha Johnson, Ian Johnston, Jon-Paul Keatley, Moritz Kraemer, Angie Lackenby, Mara Lawniczak, David Lee, Rich Livett, Stephanie Lo, Daniel Mair, Joshua Maksimovic, Nikos Manesis, Robin Manley, Carmen Manso, Angela Marchbank, Inigo Martincorena, Tamyo Mbisa, Kathryn McCluggage, J.T. McCrone, Shahjahan Miah, Michelle L. Michelsen, Mari Morgan, Gaia Nebbia, Charlotte Nelson, Jenna Nichols, Paola Niola, Kyriaki Nomikou, Steve Palmer, Naomi Park, Yasmin A. Parr, Paul J. Parsons, Vineet Patel, Minal Patel, Clare Pearson, Steven Platt, Christoph Puethe, Mike Quail, Jayna Raghwani, Lucille Rainbow, Shavanthi Rajatileka, Mary Ramsay, Paola C. Resende Silva, Steven Rudder, Chris Ruis, Christine M. Sambles, Fei Sang, Ulf Schaefer, Emily Scher, Carol Scott, Lesley Shirley, Adrian W. Signell, John Sillitoe, Christen Smith, Katherine L. Smollett, Karla Spellman, Thomas D. Stanton, David J. Studholme, Grace Taylor-Joyce, Ana P. Tedim, Thomas Thompson, Nicholas M. Thomson, Scott Thurston, Lily Tong, Gerry Tonkin-Hill, Rachel M. Tucker, Edith E. Vamos, Tetyana Vasylyeva, Joanna Warwick-Dugdale, Danni Weldon, Mark Whitehead, David Williams, Kathleen A. Williamson, Harry D. Wilson, Trudy Workman, Muhammad Yasir, Xiaoyu Yu, Alex Zarebski, Evelien M. Adriaenssens, Shazaad S.Y. Ahmad, Adela Alcolea-Medina, John Allan, Patawee Asamaphan, Laura Atkinson, Paul Baker, Jonathan Ball, Edward Barton, Mathew A. Beale, Charlotte Beaver, Andrew Beggs, Andrew Bell, Duncan J Berger, Louise Berry, Claire M. Bewshea, Kelly Bicknell, Paul Bird, Chloe Bishop, Tim Boswell, Cassie Breen, Sarah K. Buddenborg, Shirelle Burton-Fanning, Vicki Chalker, Joseph G. Chappell, Themoula Charalampous, Claire Cormie, Nick Cortes, Lindsay J. Coupland, Angela Cowell, Rose K. Davidson, Joana Dias, Maria Diaz, Thomas Dibling, Matthew J. Dorman, Nichola Duckworth, Scott Elliott, Sarah Essex, Karlie Fallon, Theresa Feltwell, Vicki M Fleming, Sally Forrest, Luke Foulser, Maria V. Garcia-Casado, Artemis Gavriil, Ryan P. George, Laura Gifford, Harmeet K. Gill, Jane Greenaway, Luke Griffith, Ana Victoria Gutierrez, Antony D. Hale, Tanzina Haque, Katherine L. Harper, Ian Harrison, Judith Heaney, Thomas Helmer, Ellen E. Higginson, Richard Hopes, Hannah C. Howson-Wells, Adam D. Hunter, Robert Impey, Dianne Irish-Tavares, David A. Jackson, Kathryn A. Jackson, Amelia Joseph, Leanne Kane, Sally Kay, Leanne M. Kermack, Manjinder Khakh, Stephen P. Kidd, Anastasia Kolyva, Jack C.D. Lee, Laura Letchford, Nick Levene, Lisa J. Levett, Michelle M. Lister, Allyson Lloyd, Joshua Loh, Louissa R. Macfarlane-Smith, Nicholas W. Machin, Mailis Maes, Samantha McGuigan, Liz McMinn, Lamia Mestek-Boukhibar, Zoltan Molnar, Lynn Monaghan, Catrin Moore, Plamena Naydenova, Alexandra S. Neaverson, Rachel Nelson, Marc O. Niebel, Elaine O’Toole, Debra Padgett, Gaurang Patel, Brendan A.I. Payne, Liam Prestwood, Veena Raviprakash, Nicola Reynolds, Alex Richter, Esther Robinson, Hazel A. Rogers, Aileen Rowan, Garren Scott, Divya Shah, Nicola Sheriff, Graciela Sluga, Emily Souster, Michael Spencer-Chapman, Sushmita Sridhar, Tracey Swingler, Julian Tang, Graham P. Taylor, Theocharis Tsoleridis, Lance Turtle, Sarah Walsh, Michelle Wantoch, Joanne Watts, Sheila Waugh, Sam Weeks, Rebecca Williams, Iona Willingham, Emma L. Wise, Victoria Wright, Sarah Wyllie, Jamie Young, Amy Gaskin, Will Rowe, Igor Siveroni, and Robert Johnson. See Data S3 for affiliation information. The members of the ISARIC4C consortia are Consortium Lead Investigator: J. Kenneth Baillie; Chief Investigator: Malcolm G. Semple; Co-Lead Investigator: Peter J.M. Openshaw; ISARIC Clinical Coordinator: Gail Carson; Co-Investigators: Beatrice Alex, Benjamin Bach, Wendy S. Barclay, Debby Bogaert, Meera Chand, Graham S. Cooke, Annemarie B. Docherty, Jake Dunning, Ana da Silva Filipe, Tom Fletcher, Christopher A. Green, Ewen M. Harrison, Julian A. Hiscox, Antonia Ying Wai Ho, Peter W. Horby, Samreen Ijaz, Saye Khoo, Paul Klenerman, Andrew Law, Wei Shen Lim, Alexander J. Mentzer, Laura Merson, Alison M. Meynert, Mahdad Noursadeghi, Shona C. Moore, Massimo Palmarini, William A. Paxton, Georgios Pollakis, Nicholas Price, Andrew Rambaut, David L. Robertson, Clark D. Russell, Vanessa Sancho-Shimizu, Janet T. Scott, Thushan de Silva, Louise Sigfrid, Tom Solomon, Shiranee Sriskandan, David Stuart, Charlotte Summers, Richard S. Tedder, Emma C. Thomson, A.A. Roger Thompson, Ryan S. Thwaites, Lance C.W. Turtle, and Maria Zambon; Project Managers: Hayley Hardwick, Chloe Donohue, Ruth Lyons, Fiona Griffiths, and Wilna Oosthuyzen; Data Analysts: Lisa Norman, Riinu Pius, Tom M. Drake, Cameron J. Fairfield, Stephen Knight, Kenneth A. Mclean, Derek Murphy, and Catherine A. Shaw; Data and Information System Managers: Jo Dalton, James Lee, Daniel Plotkin, Michelle Girvan, Egle Saviciute, Stephanie Roberts, Janet Harrison, Laura Marsh, Marie Connor, Sophie Halpin, Clare Jackson, and Carrol Gamble; Data Integration and Presentation: Gary Leeming, Andrew Law, Murray Wham, Sara Clohisey, Ross Hendry, and James Scott-Brown; Material Management: William Greenhalf, Victoria Shaw, and Sarah McDonald; Patient Engagement: Seán Keating; Outbreak Laboratory Staff and Volunteers: Katie A. Ahmed, Jane A. Armstrong, Milton Ashworth, Innocent G. Asiimwe, Siddharth Bakshi, Samantha L. Barlow, Laura Booth, Benjamin Brennan, Katie Bullock, Benjamin W.A. Catterall, Jordan J. Clark, Emily A. Clarke, Sarah Cole, Louise Cooper, Helen Cox, Christopher Davis, Oslem Dincarslan, Chris Dunn, Philip Dyer, Angela Elliott, Anthony Evans, Lorna Finch, Lewis W.S. Fisher, Terry Foster, Isabel Garcia-Dorival, Willliam Greenhalf, Philip Gunning, Catherine Hartley, Antonia Ho, Rebecca L. Jensen, Christopher B. Jones, Trevor R. Jones, Shadia Khandaker, Katharine King, Robyn T. Kiy, Chrysa Koukorava, Annette Lake, Suzannah Lant, Diane Latawiec, L. Lavelle-Langham, Daniella Lefteri, Lauren Lett, Lucia A. Livoti, Maria Mancini, Sarah McDonald, Laurence McEvoy, John McLauchlan, Soeren Metelmann, Nahida S. Miah, Joanna Middleton, Joyce Mitchell, Shona C. Moore, Ellen G. Murphy, Rebekah Penrice-Randal, Jack Pilgrim, Tessa Prince, Will Reynolds, P. Matthew Ridley, Debby Sales, Victoria E. Shaw, Rebecca K. Shears, Benjamin Small, Krishanthi S. Subramaniam, Agnieska Szemiel, Aislynn Taggart, Jolanta Tanianis-Hughes, Jordan Thomas, Erwan Trochu, Libby van Tonder, Eve Wilcock, and J. Eunice Zhang; Local Principal Investigators: Kayode Adeniji, Daniel Agranoff, Ken Agwuh, Dhiraj Ail, Ana Alegria, Brian Angus, Abdul Ashish, Dougal Atkinson, Shahedal Bari, Gavin Barlow, Stella Barnass, Nicholas Barrett, Christopher Bassford, David Baxter, Michael Beadsworth, Jolanta Bernatoniene, John Berridge, Nicola Best, Pieter Bothma, David Brealey, Robin Brittain-Long, Naomi Bulteel, Tom Burden, Andrew Burtenshaw, Vikki Caruth, David Chadwick, Duncan Chambler, Nigel Chee, Jenny Child, Srikanth Chukkambotla, Tom Clark, Paul Collini, Catherine Cosgrove, Jason Cupitt, Maria-Teresa Cutino-Moguel, Paul Dark, Chris Dawson, Samir Dervisevic, Phil Donnison, Sam Douthwaite, Ingrid DuRand, Ahilanadan Dushianthan, Tristan Dyer, Cariad Evans, Chi Eziefula, Chrisopher Fegan, Adam Finn, Duncan Fullerton, Sanjeev Garg, Sanjeev Garg, Atul Garg, Effrossyni Gkrania-Klotsas, Jo Godden, Arthur Goldsmith, Clive Graham, Elaine Hardy, Stuart Hartshorn, Daniel Harvey, Peter Havalda, Daniel B. Hawcutt, Maria Hobrok, Luke Hodgson, Anil Hormis, Michael Jacobs, Susan Jain, Paul Jennings, Agilan Kaliappan, Vidya Kasipandian, Stephen Kegg, Michael Kelsey, Jason Kendall, Caroline Kerrison, Ian Kerslake, Oliver Koch, Gouri Koduri, George Koshy, Shondipon Laha, Steven Laird, Susan Larkin, Tamas Leiner, Patrick Lillie, James Limb, Vanessa Linnett, Jeff Little, Michael MacMahon, Emily MacNaughton, Ravish Mankregod, Huw Masson, Elijah Matovu, Katherine McCullough, Ruth McEwen, Manjula Meda, Gary Mills, Jane Minton, Mariyam Mirfenderesky, Kavya Mohandas, Quen Mok, James Moon, Elinoor Moore, Patrick Morgan, Craig Morris, Katherine Mortimore, Samuel Moses, Mbiye Mpenge, Rohinton Mulla, Michael Murphy, Megan Nagel, Thapas Nagarajan, Mark Nelson, Igor Otahal, Mark Pais, Selva Panchatsharam, Hassan Paraiso, Brij Patel, Natalie Pattison, Justin Pepperell, Mark Peters, Mandeep Phull, Stefania Pintus, Jagtur Singh Pooni, Frank Post, David Price, Rachel Prout, Nikolas Rae, Henrik Reschreiter, Tim Reynolds, Neil Richardson, Mark Roberts, Devender Roberts, Alistair Rose, Guy Rousseau, Brendan Ryan, Taranprit Saluja, Aarti Shah, Prad Shanmuga, Anil Sharma, Anna Shawcross, Jeremy Sizer, Manu Shankar-Hari, Richard Smith, Catherine Snelson, Nick Spittle, Nikki Staines, Tom Stambach, Richard Stewart, Pradeep Subudhi, Tamas Szakmany, Kate Tatham, Jo Thomas, Chris Thompson, Robert Thompson, Ascanio Tridente, Darell Tupper-Carey, Mary Twagira, Andrew Ustianowski, Nick Vallotton, Lisa Vincent-Smith, Shico Visuvanathan, Alan Vuylsteke, Sam Waddy, Rachel Wake, Andrew Walden, Ingeborg Welters, Tony Whitehouse, Paul Whittaker, Ashley Whittington, Meme Wijesinghe, Martin Williams, Lawrence Wilson, Sarah Wilson, Stephen Winchester, Martin Wiselka, Adam Wolverson, Daniel G. Wooton, Andrew Workman, Bryan Yates, and Peter Young.

## Supplemental information

Document S1. Tables S1–S6

Data S1. Binding, competition, and neutralization data for mAbs and sera, related to Figures 6 and 7

Data S2. GISAID acknowledgment table

Data S3. COG-UK consortium names and affiliations
