# Supplementary material for: Circulating SARS-CoV-2 spike N439K variants maintain fitness while evading antibody-mediated immunity
Source: Cell. 2021 Mar 4;184(5):1171–1187.e20. doi: 10.1016/j.cell.2021.01.037 (PMC7843029; doi:10.1016/j.cell.2021.01.037)
Supplement: Document S1. Tables S1–S6 [file mmc1.pdf]

Cell, Volume *184*

Supplemental information

Circulating SARS-CoV-2 spike N439K variants maintain fitness while evading antibody-mediated immunity

Emma C. Thomson, Laura E. Rosen, James G. Shepherd, Roberto Spreafico, Ana da Silva Filipe, Jason A. Wojcechowskyj, Chris Davis, Luca Piccoli, David J. Pascall, Josh Dillen, Spyros Lytras, Nadine Czudnochowski, Rajiv Shah, Marcel Meury, Natasha Jesudason, Anna De Marco, Kathy Li, Jessica Bassi, Aine O'Toole, Dora Pinto, Rachel M. Colquhoun, Katja Culap, Ben Jackson, Fabrizia Zatta, Andrew Rambaut, Stefano Jaconi, Vattipally B. Sreenu, Jay Nix, Ivy Zhang, Ruth F. Jarrett, William G. Glass, Martina Beltramello, Kyriaki Nomikou, Matteo Pizzuto, Lily Tong, Elisabetta Cameroni, Tristan I. Croll, Natasha Johnson, Julia Di Iulio, Arthur Wickenhagen, Alessandro Ceschi, Aoife M. Harbison, Daniel Mair, Paolo Ferrari, Katherine Smollett, Federica Sallusto, Stephen Carmichael, Christian Garzoni, Jenna Nichols, Massimo Galli, Joseph Hughes, Agostino Riva, Antonia Ho, Marco Schiuma, Malcolm G. Semple, Peter J.M. Openshaw, Elisa Fadda, J. Kenneth Baillie, John D. Chodera, The ISARIC4C Investigators, the COVID-19 Genomics UK (COG-UK) Consortium, Suzannah J. Rihn, Samantha J. Lycett, Herbert W. Virgin, Amalio Telenti, Davide Corti, David L. Robertson, and Gyorgy Snell

Table S1. Details of the *Sarbecovirus* sequences related to STAR Methods section "RBM variability across *Sarbecoviruses*" and Figure S2. The top 8 sequences shaded in gray were used for the % identity sliding window plot and all 69 sequences were used for the entropy plot.

Virus	Host	Collection place	Accession	Collection time	GISAID acknowledgments
Wuhan-Hu-1|SARS-CoV-2	human	Wuhan	MN908947	2020-12-19	
WIV1	Bat-R_sinicus	Yunnan-Kunming	KF367457	2020-09-12	
HSZ-Cc|SARS-CoV-1	human	Guangzhou	AY394995	1905-06-24	
RaTG13	Bat-R_affinis	Yunnan	EPI_ISL_402131	2013-07-24	Yan Zhu; Ping Yu; Bei Li; Ben Hu; Hao-Rui Si; Xing-Lou Yang; Peng Zhou; Zheng-Li Shi
GD Pangolin	pangolin	Guandong	EPI_ISL_410721	2020-02-16	Yongyi Shen; Lihua Xiao; Wu Chen
GX Pangolin	pangolin	Guangxi	EPI_ISL_410539	1905-07-09	Wu-Chun Cao; Tommy Tsan-Yuk Lam; Na Jia; Ya-Wei Zhang; Jia-Fu Jiang; Bao-Gui Jiang
BtKY72	Bat-R_spp	Kenya	KY352407	2020-10-07	
BM48-31	Bat-R_blasii	Bulgaria	NC_014470	2020-04-08	
P4L	pangolin	Guangxi	EPI_ISL_410538	1905-07-09	Wu-Chun Cao; Tommy Tsan-Yuk Lam; Na Jia; Ya-Wei Zhang; Jia-Fu Jiang; Bao-Gui Jiang
P5L	pangolin	Guangxi	EPI_ISL_410540	1905-07-09	Wu-Chun Cao; Tommy Tsan-Yuk Lam; Na Jia; Ya-Wei Zhang; Jia-Fu Jiang; Bao-Gui Jiang
P5E	pangolin	Guangxi	EPI_ISL_410541	1905-07-09	Wu-Chun Cao; Tommy Tsan-Yuk Lam; Na Jia; Ya-Wei Zhang; Jia-Fu Jiang; Bao-Gui Jiang
P2V	pangolin	Guangxi	EPI_ISL_410542	1905-07-09	Wu-Chun Cao; Tommy Tsan-Yuk Lam; Na Jia; Ya-Wei Zhang; Jia-Fu Jiang; Bao-Gui Jiang
RmYN02	Bat-R_malayanus	Yunnan-Xishuangbanna	EPI_ISL_412977	2019-06-25	Weifeng Shi; Tao Hu; Hong Zhou; Juan Li; Xing Chen; Alice Catherine Hughes; Yuhai Bi
RpShaanxi2011	Bat-R_pusillus	Shaanxi	JX993987	2020-09-11	
HuB2013	Bat-R_sinicus	Hubei	KJ473814	2020-04-13	
GX2013	Bat-R_sinicus	Guangxi	KJ473815	2020-11-12	
Rs4231	Bat-R_sinicus	Yunnan-Kunming	KY417146	2013-04-17	
Rs4255	Bat-R_sinicus	Yunnan-Kunming	KY417149	2013-04-17	
Rs4237	Bat-R_sinicus	Yunnan-Kunming	KY417147	2013-04-17	
Rs4247	Bat-R_sinicus	Yunnan-Kunming	KY417148	2013-04-17	
YN2018B	Bat-R_affinis	Yunnan	MK211376	2020-09-16	
YN2018D	Bat-R_affinis	Yunnan	MK211378	2020-09-16	
Rs7327	Bat-R_sinicus	Yunnan--Kunming	KY417151	2014-10-24	
Rs9401	Bat-R_sinicus	Yunnan-Kunming	KY417152	2015-10-16	
RsSHC014	Bat-R_sinicus	Yunnan-Kunming	KC881005	2011-04-17	
Rs3367	Bat-R_sinicus	Yunnan-Kunming	KC881006	2012-03-19	
YN2018C	Bat-R_affinis	Yunnan-Kunming	MK211377	2020-09-16	
As6526	Bat-Aselliscus_stoliczkanus	Yunnan-Kunming	KY417142	2014-05-12	
Rs4081	Bat-R_sinicus	Yunnan-Kunming	KY417143	2012-09-18	
Anlong-103	Bat-R_sinicus	Guizhou-Anlong	KY770858	1905-07-05	
Anlong-112	Bat-R_sinicus	Guizhou-Anlong	KY770859	1905-07-05	
YN2018A	Bat-R_affinis	Yunnan	MK211375	2020-09-16	
YN2013	Bat-R_sinicus	Yunnan	KJ473816	2020-12-10	
WIV16	Bat-R_sinicus	Yunnan-Kunming	KT444582	2013-07-21	
Rs4874	Bat-R_sinicus	Yunnan-Kunming	KY417150	2013-07-21	
Rf4092	Bat-R_ferrumequinum	Yunnan-Kunming	KY417145	2012-09-18	
F46	Bat-R_pusillus	Yunnan	KU973692	1905-07-04	
Rs672	Bat-R_sinicus	Guizhou	FJ588686	2020-09-06	
Rs4084	Bat-R_sinicus	Yunnan-Kunming	KY417144	2012-09-18	
Rp3	Bat-R_pearsoni	Guangxi-Nanning	DQ071615	2020-12-04	
YNLF_31C	Bat-R_ferrumequinum	Yunnan-Lufeng	KP886808	2013-05-23	
YNLF_34C	Bat-R_ferrumequinum	Yunnan-Lufeng	KP886809	2013-05-23	
LYRa11	Bat-R_affinis	Yunnan-Baoshan	KF569996	1905-07-03	
HKU3-1	Bat-R_sinicus	Hong_Kong	DQ022305	2005-02-17	
HKU3-3	Bat-R_sinicus	Hong_Kong	DQ084200	2005-03-17	
HKU3-2	Bat-R_sinicus	Hong_Kong	DQ084199	2005-02-24	
HKU3-5	Bat-R_sinicus	Hong_Kong	GQ153540	2005-09-20	
HKU3-10	Bat-R_sinicus	Hong_Kong	GQ153545	2006-10-28	
HKU3-9	Bat-R_sinicus	Hong_Kong	GQ153544	2006-10-28	
HKU3-11	Bat-R_sinicus	Hong_King	GQ153546	2007-03-07	
HKU3-13	Bat-R_sinicus	Hong_Kong	GQ153548	2007-11-15	
HKU3-6	Bat-R_sinicus	Hong_Kong	GQ153541	2005-12-16	
HKU3-4	Bat-R_sinicus	Hong_Kong	GQ153539	2005-07-20	
HKU3-12	Bat-R_sinicus	Hong_Kong	GQ153547	2007-05-15	
HKU3-7	Bat-R_sinicus	Guangdong	GQ153542	2006-02-15	
HKU3-8	Bat-R_sinicus	Guangdong	GQ153543	2006-02-15	
HeB2013	Bat-R_ferrumequinum	Hebei	KJ473812	2020-04-13	
Jiyuan-84	Bat-R_ferrumequinum	Henan-Jiyuan	KY770860	1905-07-04	
SX2013	Bat-R_ferrumequinum	Shanxi	KJ473813	2020-11-13	
Rf1	Bat-R_ferrumequinum	Hubei-Yichang	DQ412042	2020-11-04	
SC2018	Bat-R_spp	Sichuan	MK211374	2020-10-16	
Yunnan2011	Bat-Chaerephon_plicata	Yunnan	JX993988	2020-11-11	
Longquan_140	Bat-R_monoceros	China	KF294457	1905-07-04	
JL2012	Bat-R_ferrumequinum	Jilin	KJ473811	2179-04-18	
JTMC15	Bat-R_ferrumequinum	Jilin	KU182964	2020-10-13	
279_2005	Bat-R_macrotis	Hubei	DQ648857	2020-11-04	
Rm1	Bat-R_macrotis	Hubei	DQ412043	2020-11-04	
CoVZC45	Bat-R_sinicus	Zhoushan-Dinghai	MG772933	2020-02-17	
CoVZXC21	Bat-R_sinicus	Zhoushan-Dinghai	MG772934	2020-07-15	

Table S2. Sequence counts by country, related to Figure 3

N439K variant counts and numbers of SARS-CoV-2 genomes sequenced by country were retrieved from GISAID on January 6, 2021. Cumulative confirmed positive cases were retrieved from the WHO Coronavirus Disease (COVID-19) Dashboard (https://covid19.who.int) and for the UK countries from UK government data (https://coronavirus.data.gov.uk/details/cases) to January 6, 2021. N439K % sequenced = N439K counts divided by total genomes sequenced. Genomes sequenced % positives = total genomes sequenced divided by confirmed positive cases for each country. N439K estimated positive cases = % N439K sequenced multiplied by confirmed positive cases.

	N439K		Genomes sequenced		Confirmed positive cases	
**Country**	**counts**	**% sequenced**	**counts**	**% positives**	**total reported**	**N439K estimated**
**Australia**	8	0.05	16604	58.22	28519	14
**Belgium**	25	0.85	2930	0.45	654352	5583
**Bosnia_and_Herzegovina**	1	3.03	33	0.03	112828	3419
**Brazil**	2	0.11	1824	0.02	7753752	8502
**Croatia**	3	6.00	50	0.02	214390	12863
**Czech_Republic**	107	37.94	282	0.04	759635	288230
**Denmark**	2307	10.39	22212	13.01	170787	17738
**England**	2412	2.13	113052	4.61	2450983	52292
**Faroe_Islands**	7	18.92	37	5.95	622	118
**Finland**	1	0.11	906	2.45	36919	41
**France**	26	0.89	2920	0.11	2615185	23286
**Germany**	84	4.23	1984	0.11	1787410	75677
**Hungary**	1	0.37	273	0.08	329721	1208
**Ireland**	188	13.32	1411	1.31	107997	14389
**Italy**	28	1.97	1419	0.07	2166244	42745
**Japan**	9	0.09	9650	3.82	252317	235
**Luxembourg**	28	1.39	2019	4.30	46985	652
**Morocco**	1	1.12	89	0.02	445439	5005
**Netherlands**	71	1.58	4484	0.54	827622	13105
**New_Zealand**	1	0.09	1063	58.15	1828	2
**Nigeria**	1	0.45	224	0.25	91284	408
**Northern_Ireland**	36	1.68	2139	2.57	83236	1401
**Norway**	38	4.51	842	1.66	50715	2289
**Poland**	28	8.28	338	0.03	1330543	110222
**Portugal**	4	0.18	2269	0.53	431623	761
**Romania**	7	4.70	149	0.02	643559	30234
**Scotland**	768	6.03	12726	9.02	141066	8513
**Singapore**	6	0.38	1578	2.69	58749	223
**Slovakia**	3	8.11	37	0.02	191088	15494
**South_Korea**	4	0.31	1287	1.96	65815	205
**Sweden**	31	2.61	1187	0.26	462767	12086
**Switzerland**	101	2.03	4973	1.08	459660	9336
**USA**	9	0.02	59063	0.29	20643544	3146
**Wales**	522	2.78	18791	11.63	161516	4487
**Total N439K = (observed)**	6868				**Total N439K = (estimated)**	763907

Table S3. Crystallographic data collection and refinement statistics, related to Figure 4. Values in parentheses are for highest-resolution shell.

	RBD/S304/S309/ACE2
**PDB ID**	7L0N
**Data collection**	
X-ray source	ALS BL4.2.2
Space group	$P2_1$
Cell dimensions	
a, b, c (Å)	78.0, 186.6, 194.5
α, β, γ (°)	90, 96.4, 90
Resolution (Å)	48.95-2.78 (2.83-2.78)
R_{sym} or R_{merge}	0.47 (3.03)
$CC_{1/2}$	0.97 (0.18)
I / σI	3.3 (0.5)
Completeness (%)	94.8 (99.8)
Redundancy	7.2 (4.7)
**Refinement**	
Resolution (Å)	2.78
No. reflections	122372
R_{work} / R_{free}	0.292/0.325
No. atoms	26477
Mean B-factors (Å^2)	86.1
R.m.s. deviations	
Bond lengths (Å)	0.002
Bond angles (°)	0.668

Table S4. Viral load analysis, related to Figure 5. Parameter estimates on the link scale from the model estimating the impact of the N439K mutation on the Ct value of patients infected with SARS-CoV-2 in Scotland. Credible intervals represent 95% the shortest posterior density intervals. The difference between D614G/N439 and D614G/N439K was estimated by direct subtraction of the Hamiltonian Monte Carlo samples of the D614G/N439K estimate from the D614G/N439 estimate. Ct value did not appear strongly correlated with biological sex or age after controlling for the other factors. Patients infected with related viral genomes had correlated Ct values at testing potentially implying that there are other undescribed mutations in the genome that are affecting the viral load.

	Posterior mean	Lower bound of 95% shortest probability interval	Upper bound of 95% shortest probability interval
D614G/N439	-0.31	-1.91	1.48
D614G/N439K	-0.96	-2.68	0.74
Female biological sex	0.05	-0.34	0.43
Intercept	27.22	26.48	27.97
Cluster random effect standard deviation	0.92	0.00	1.49
Phylogenetic random effect standard deviation	0.99	0.30	1.72
Age penalised spline standard deviation	1.15	0.00	3.15
Days since first case in the dataset penalised spline standard deviation	9.78	4.42	16.22
Residual standard deviation	3.91	3.71	4.12
Difference between D614G/N439 and D614G/N439K	-0.65	-1.22	-0.07

Table S5. Clinical severity analysis, related to Figure 5. Parameter estimates on the link scale from the model estimating the impact of the N439K mutation on the severity of infection of patients infected with SARS-CoV-2 in Scotland. Credible intervals represent 95% the shortest posterior density intervals. Thresholds correspond to the positions of the boundaries between the different severity classes.

	Posterior mean	Lower bound of 95% shortest probability interval	Upper bound of 95% shortest probability interval
Presence of 614G	0.06	-1.21	1.33
Presence of 614G and 439K	0.23	-1.01	1.56
Female biological sex	-0.79	-1.02	-0.55
Threshold 1	-0.02	-0.55	0.54
Threshold 2	0.87	0.33	1.43
Threshold 3	1.23	0.68	1.78
Cluster random effect standard deviation	0.30	0.00	0.62
Phylogenetic random effect standard deviation	0.92	0.34	1.49
Age penalised spline standard deviation	1.31	0.09	2.85
Days since first case in the dataset penalised spline standard deviation	3.03	0.81	5.36

Table S6. Viral isolate genotypes, related to Figure 5. Nucleotide differences between GLA1 and GLA2. SNPs determined by CoV-GLUE on consensus sequences relative to Wuhan-Hu-1 (NC_045512.2).

Sample	SNP	Amino Acid Change	
		Gene	Mutation
GLA1	C3037T		
	C14408T	nsp12	P323L
	A23403G	S	D614G
	A24388T		
	T26258C	E	V5A
GLA2	C3037T		
	C14408T	nsp12	P323L
	T19724C	nsp15	V35A
	C22879A	S	N439K
	A23403G	S	D614G
	G29573T	ORF 10	V6F
